# Population whole-genome bisulfite sequencing across two tissues highlights the environment as the principal source of human methylome variation

**DOI:** 10.1186/s13059-015-0856-1

**Published:** 2015-12-23

**Authors:** Stephan Busche, Xiaojian Shao, Maxime Caron, Tony Kwan, Fiona Allum, Warren A. Cheung, Bing Ge, Susan Westfall, Marie-Michelle Simon, The Multiple Tissue Human Expression Resource, Amy Barrett, Jordana T. Bell, Mark I. McCarthy, Panos Deloukas, Mathieu Blanchette, Guillaume Bourque, Timothy D. Spector, Mark Lathrop, Tomi Pastinen, Elin Grundberg

**Affiliations:** Department of Human Genetics, McGill University, 740 Dr. Penfield Avenue, H3A 0G1 Montreal, Quebec Canada; McGill University and Genome Quebec Innovation Centre, Montreal, Quebec Canada; Oxford Centre for Diabetes, Endocrinology & Metabolism, University of Oxford, Churchill Hospital, Headington, Oxford, UK; Department of Twin Research and Genetic Epidemiology, King’s College London, London, UK; Wellcome Trust Centre for Human Genetics, University of Oxford, Oxford, UK; Oxford National Institute for Health Research Biomedical Research Centre, Churchill Hospital, Headington, Oxford, UK; William Harvey Research Institute, Queen Mary University of London, London, UK; Wellcome Trust Sanger Institute, Hinxton, Cambridge, UK; School of Computer Science, McGill University, Montreal, Quebec Canada

**Keywords:** Adipose, Blood, CpH methylation, Differentially methylated region (DMR), DNA methylation, Enhancer, Environment, Population, Twins, Whole-genome bisulfite sequencing (WGBS)

## Abstract

**Background:**

CpG methylation variation is involved in human trait formation and disease susceptibility. Analyses within populations have been biased towards CpG-dense regions through the application of targeted arrays. We generate whole-genome bisulfite sequencing data for approximately 30 adipose and blood samples from monozygotic and dizygotic twins for the characterization of non-genetic and genetic effects at single-site resolution.

**Results:**

Purely invariable CpGs display a bimodal distribution with enrichment of unmethylated CpGs and depletion of fully methylated CpGs in promoter and enhancer regions. Population-variable CpGs account for approximately 15–20 % of total CpGs per tissue, are enriched in enhancer-associated regions and depleted in promoters, and single nucleotide polymorphisms at CpGs are a frequent confounder of extreme methylation variation. Differential methylation is primarily non-genetic in origin, with non-shared environment accounting for most of the variance. These non-genetic effects are mainly tissue-specific. Tobacco smoking is associated with differential methylation in blood with no evidence of this exposure impacting cell counts. Opposite to non-genetic effects, genetic effects of CpG methylation are shared across tissues and thus limit inter-tissue epigenetic drift. CpH methylation is rare, and shows similar characteristics of variation patterns as CpGs.

**Conclusions:**

Our study highlights the utility of low pass whole-genome bisulfite sequencing in identifying methylome variation beyond promoter regions, and suggests that targeting the population dynamic methylome of tissues requires assessment of understudied intergenic CpGs distal to gene promoters to reveal the full extent of inter-individual variation.

**Electronic supplementary material:**

The online version of this article (doi:10.1186/s13059-015-0856-1) contains supplementary material, which is available to authorized users.

## Background

The human genome comprises ~600 million cytosine bases on each strand, with ~5 % in the CpG dinucleotide context. Methylation of Cs in this context is a common epigenetic modification classically studied as a silencing mark when occurring in promoter regions or CpG islands (CGIs) [1]. Other general findings on methylation and gene regulation are gene body methylation showing positive correlation with expression [2] and exon/intron boundary methylation level differences correlating with alternative splicing [3].

CpG methylation has been intensively investigated on a genome-wide level for association with clinical phenotypes [4]. These studies have identified specific differentially methylated regions (DMRs) associated with various diseases, including cancer [5, 6], multiple sclerosis [7], Alzheimer’s disease [8], rheumatoid arthritis [9], and immunoglobulin E concentration in association with allergic diseases [10]. Variation in CpG methylation has also been investigated in healthy populations using array-based profiling (e.g., Illumina HumanMethylation450 Beadchip, hereafter Illumina 450 K array) of large population-based cohorts, associating differential CpG methylation in blood with metabolic traits [11] and body mass index [12], and in CD4+ T-cells with fasting lipid levels [13]. Similarly, we recently utilized the Illumina 450 K array to assess methylation variation across 648 adipose samples from twins belonging to the MuTHER cohort and observed overall low variance in methylation across healthy individuals [14]. In addition, we showed that methylation signatures in enhancer elements exhibit a more pronounced pattern of inter-individual variation compared to promoter regions.

However, a main limitation with studies using the Illumina 450 K array is that the platform only covers ~1.5 % of overall genomic CpGs, which are biased towards promoters and strongly underrepresented in distal regulatory elements, i.e., enhancers. Compared to targeted array-based methods, whole-genome bisulfite sequencing (WGBS) offers single-site resolution CpG methylation interrogation at full genomic coverage. Recent sequencing-based studies across multiple developmental and somatic mouse and human cell types identified only ~20 % of CpGs as variable [15–17]. In these studies, DMRs across tissues were also shown to mainly map outside of promoter-associated regions (mostly outside regions covered by the 450 K array) and to be highly enriched in enhancers.

Another advantage of WGBS is its ability to access patterns of non-CpG or CpH (H = A, C, T) methylation. CpH methylation is asymmetric, meaning that methylation is often observed on only one strand. Previous studies have identified that CpH methylation accounts for ~25 % of all methylated cytosines in human embryonic stem cells [2], ~35 % in brain tissue [18], and ~65 % in oocytes [19], but is rare in differentiated somatic cells like fibroblasts and monocytes [2, 3]. However, despite its low frequency, some functional relevance is thought to be associated with CpH methylation in somatic cells outside the brain [20].

As highlighted above, most genome-wide methylation studies of inter-individual variation to date have been biased towards promoter and CpG-dense regions. Comprehensive and unbiased analyses of population variation at single-CpG/CpH resolution have not been carried out so far. In a recent study, Ziller et al. applied down-sampling analysis to NIH Roadmap data generated in healthy cells and tissues with 30-fold coverage and discovered that per-sample coverage of 5–15-fold is sufficient for the identification of DMRs [21].

To address this gap and to explore the contribution of genetic versus non-genetic factors to global methylome variation, we applied WGBS to generate full genome single-site resolution methylomes of 34 adipose and 27 blood samples from monozygotic (MZ) and dizygotic (DZ) twins of the MuTHER cohort [22]. Our per-sample mean genome coverage of ~7-fold interrogated the methylation state of ~25 million CpGs and ~1 billion CpHs. Through thorough quality control and filtering steps, we characterized the global DNA methylation landscape, including frequency and genome feature association of population static and dynamic CpG and CpH methylation. By taking advantage of the twin structure, we estimated the genetic versus environmental origin (shared and non-shared/unique) of variation and developed a method to detect population differentially methylation regions (pDMRs), which we distinguish from those of non-shared environmental origin (eDMRs). Finally, we associated these pDMRs and eDMRs with functional relevance by performing transcription factor (TF) binding site motif and pathway analysis, and by investigating expression of associated genes in adipocytes and hematopoietic cells.

## Results

### Study cohort, and data generation and processing

We performed WGBS on 34 adipose (seven MZ pairs, six DZ pairs, and eight singletons) and 27 blood (seven MZ pairs, six DZ pairs, and one singleton) DNA samples derived from a total of 43 female twins belonging to the MuTHER cohort [22, 23] (see “Methods” and Additional file 1: Table S1). We generated 11.5 billion 100 base pair (bp) paired-end reads covering 2.3 tera base pairs (Tbp) of sequence. We applied standard alignment methods and filters to obtain a mean genome coverage of 6.3-fold (range 1.0-fold to 12.9-fold) for adipose and 8.7-fold (range 0.7-fold to 29.0-fold) for blood (Additional file 1: Table S1). We compared the mean genome coverage to the number of overall detected CpGs for each sample, observing that CpG-discovery was saturated at ~6-fold coverage, detecting ~27 million sites (Additional file 2: Figure S1). The median bisulfite conversion efficiency of CpHs was determined to be 99.4 % (range 97.4–99.8 %; Additional file 1: Table S1).

**Fig. 1:**
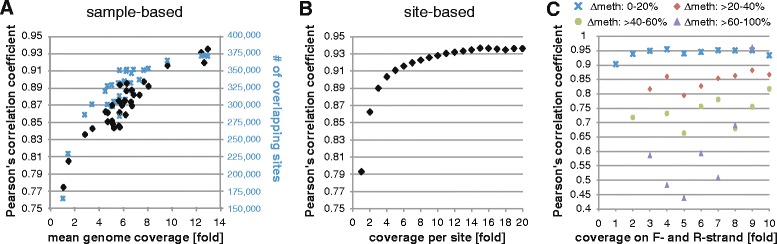
Comparison of sequencing-derived and array-derived methylation data. 450 K methylation array data were available for adipose tissue, and methylation values for CpG-sites jointly interrogated by both whole-genome bisulfite sequencing and the array were extracted for each individual. Shown are Pearson’s correlation coefficients derived from comparing array to sequencing methylation data for jointly interrogated CpG-sites for (**a**) samples at indicated mean genome coverage (*black diamonds*), with *blue crosses* indicating the number of jointly detected sites per sample, (**b**) CpG-sites at indicated coverage across all samples, (**c**) CpG-sites displaying 0 to 20 % (*blue crosses*), >20 to 40 % (*red diamonds*), >40 to 60 % (*green points*), or >60–100 % (*purple triangles*) methylation difference between forward and reverse strands at indicated sequencing coverage

We have previously profiled the adipose samples on the Illumina 450 K array [14], which we used here for comparison. We observed sample-based correlations of Illumina 450 K and WGBS to saturate at 10-fold to 12-fold average genome sequencing coverage (Pearson’s R ~0.94) (Fig. 1a), and at 12-fold per CpG coverage (Pearson’s R ~ 0.94) for CpG-based correlations (Fig. 1b). Additionally, we observed correlation to be highly dependent on strand-concordance of methylation, i.e., we observed low correlation of CpGs displaying discordant methylation between forward and reverse strands (Fig. 1c). We also noted low correlation across the two approaches for CpGs displaying abnormal high coverage (see “Methods”). These CpGs were shown to be enriched in “blacklisted” regions defined by the ENCODE project (http://hgwdev.cse.ucsc.edu/cgi-bin/hgFileUi?db=hg19&g=wgEncodeMapability), which include genomic regions with artifactual high read counts across tissues and cell lines in DNaseI, formaldehyde-assisted isolation of regulatory elements (FAIRE), and ChIP-seq experiments [24]. Taken together, in all subsequent analysis we excluded CpGs not covered by at least two reads per strand with an absolute strand difference in methylation of ≤ 20 %, CpGs located within blacklisted regions defined by ENCODE or us (see “Methods”), and CpGs not located on autosomes, leaving on average 7.9 × 10^6^ unique and high-confidence CpGs per sample. The number of CpGs passing all filters correlated well with overall sequencing depth with a ratio of approximately 1 million detected CpGs per 1-fold mean coverage, saturating at ~20-fold coverage (Additional file 2: Figure S1).

### Global CpG methylation patterns

First, we aimed to characterize the global CpG methylation landscape per tissue and thus combined all datasets. In total, we covered 23.6 × 10^6^ and 25.0 × 10^6^ CpGs in adipose and blood, respectively, with a mean methylation of 80 % irrespective of tissue (Additional file 2: Figure S2A, B). Because private sequence variants mapping to CpGs (i.e., introducing or removing) might bias methylation measures, we overlapped the two datasets with dbSNP137 [25] and noted that as much as 27 % of our detected CpGs in fact overlapped an annotated single nucleotide polymorphism (SNP). Taking this into account, we covered 17.2 × 10^6^ and 18.4 × 10^6^ CpGs (Additional file 1: Table S2) in the combined variant-removed adipose and blood datasets, respectively. We noted that removing SNPs only marginally impacted mean and median methylation levels (Additional file 2: Figure S2C, D). Of these CpGs, 19 % and 22 % mapped to CGI-associated regions in adipose and blood, respectively, leaving the vast majority mapping to regions with lower CpG density. Focusing on the genic context we observed ~53 % of CpGs per tissue to locate within gene regions (Additional file 1: Table S2; for region annotation see “Methods”).

**Fig. 2:**
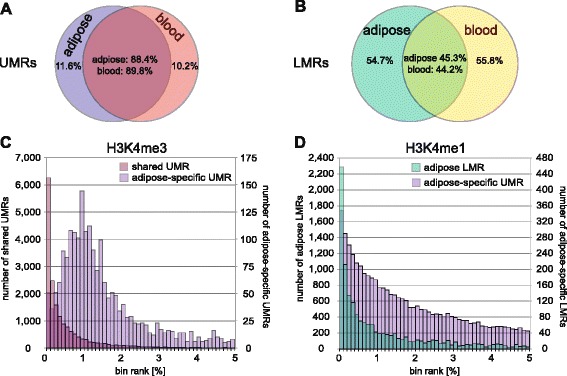
DNA methylation footprint in adipose tissue and blood. Datasets were merged within tissues, keeping only sites covered ≥12-fold, and the MethylseekR software [26] was employed to identify unmethylated and low-methylated footprints in adipose and blood. Shown are Venn diagrams describing tissue-specificity of identified (**a**) unmethylated regions (*UMR*) and (**b**) low-methylated regions (*LMR*). For overlapping areas, numbers indicate percentage of overlap based on adipose tissue and blood as indicated. **c** Histograms display the number of tissue-shared and adipose-specific UMRs overlapping with ranked H3K4me3 bins (see text; top 5 % are shown). **d** Adipose LMRs and adipose-specific UMRs overlapped with ranked H3K4me1 bins (top 5 % are shown)

Previous studies have identified areas with low CpG methylation (<50 %) to comprise active regulatory regions [17, 26, 27], which we aimed to study in more detail here using the combined datasets. To be conservative we only kept CpGs with a minimum coverage of 12-fold (14.7 × 10^6^ CpGs in adipose, 17.8 × 10^6^ CpGs in blood). We identified on average 63,000 low-methylated regions per tissue with ~30 % being unmethylated regions and the remainder being low-methylated regions (Additional file 1: Table S3A). Irrespective of tissue, unmethylated regions displayed stable methylation across regions nearing 0 % and spanning an average genomic size of ~2400 bp and on average 122 CpGs per region. In contrast, low-methylated regions displayed methylation levels between 5 % and 45 % and a size of ~750 bp (Additional file 1: Table S3B, Additional file 2: Figure S3A, B) with only on average 11 CpGs. We then compared these different unmethylated and low-methylated regions across tissues and found that as much as 90 % of the detected unmethylated regions were identified in both tissues, compared to the low-methylated regions that displayed more tissue-specificity with an overlap across tissues of only ~45 % (Fig. 2a, b) [15–17].

To further investigate the characteristics of the identified adipose unmethylated and low-methylated regions, we used ChIP-seq data from the NIH Roadmap consortium [28] for the active promoter-associated H3K4me3 [29] and the active enhancer-associated H3K4me1 [30] marks in human adipocytes. We generated a normalized signal intensity ranked list for these histone marks using a background-subtracted binning approach (see “Methods” and [14]) and overlapped the top 1 % bins with the unmethylated and low-methylated adipose regions identified in the combined dataset. In total, 78 % of the adipose unmethylated regions overlapped an the H3K4me3 bin and this co-localization was further strengthened when restricting to similar regions also identified in blood, corresponding to 84 % (Fig. 2c). This pattern was further pronounced if we restricted to H3K4me3 bins located 1 kb upstream of a transcription start site (TSS) where 91 % of the adipose unmethylated regions overlapped. We also looked specifically at the unmethylated regions detected only in adipose (N = 2,320) and noted here that only 35 % overlapped a H3K4me3 bin (Fig. 2c), but instead the majority (56 %) of these overlapped with a top 1 % H3K4me1 bin, indicating these regions to be more enhancer-like (Fig. 2d). Focusing on the low-methylated regions in adipose instead, we observed enrichment in the top-ranking H3K4me1 bins (Fig. 2d), with 25 % of shared and 26 % of adipose-specific regions overlapping with the top 1 % of H3K4me1 bins. However, this overall lower enrichment of low-methylated regions in H3K4me1 bins may lie in the lower specificity of the enhancer-associated mark being more generally associated with gene activity and also localizing, e.g., to promoters [30]. Indeed, we observed low methylated regions to be enriched in the top ranking H3K4me1 bins when compared to the percentage of overlap in bins ranking lower than the top 1 % (Additional file 2: Figure S3C).

### The invariable CpG landscape

Our recent survey of methylation variation in the complete MuTHER adipose cohort (N = 648) using the Illumina 450 K array indicated that the majority of targeted CpGs show low variability in methylation levels [14]. However, whether this low variability included also purely invariable methylation states of CpGs was left unexplained owing to the technical limitations of array-based methods. We thus aimed here to study this in greater detail using our variant-removed datasets to look for constitutively unmethylated (0 %) or fully methylated (100 %) CpGs. We defined population invariable CpGs as being detected in two or more individuals per tissue and displaying methylation value standard deviation (SD) of zero across individuals. We determined invariable CpGs for each tissue and distinguished between tissue-specific (methylation SD = 0 in one and SD > 0 in the other tissue) and shared (methylation SD = 0 in both tissues). We detected 26 % and 6 % of CpGs to be invariable in adipose and blood, respectively, highlighting that invariability in DNA methylation is more frequent in adipose than in blood. This ratio changed only slightly at higher CpG sequence coverage (Additional file 1: Table S4), excluding the possibility that lower average genomic coverage in adipose leads to the discovery of fewer variant sites and indicating that higher cellular heterogeneity of blood drives variance. Most invariable CpGs in adipose were tissue-specific (10 % were shared with blood), whereas the opposite was seen in blood (i.e., 80 % of invariable CpGs in blood were also invariable in adipose). Fully methylated CpGs (100 %) comprised 80 % of all invariable CpGs within adipose, 35 % within blood, 85 % of adipose-specific, 65 % of blood-specific, and 36 % of shared total invariable CpGs (Additional file 2: Figure S4A), identifying tissue-specific invariable CpGs to be mainly methylated whereas shared CpGs are predominantly unmethylated. The lower frequency of methylated CpGs in blood could be a reflection of greater cellular diversity compared to adipose tissue.

We also investigated the distribution of unmethylated (0 %) and fully methylated (100 %) invariable CpGs within genomic features by overlapping with CGI features, genic regions (see “Methods”), and with NIH Roadmap H3K4me1 and H3K4me3 ChIP-seq data (available for adipose only). Compared to background (all CpGs detected in two or more individuals), unmethylated CpGs in adipose displayed a 12-fold to 14-fold significant (Fisher’s exact test *p* < 1.0 × 10^-16^) enrichment in promoter-associated regions (CGIs, TSS200, exon 1, H3K4me3), and slighter 3.5-fold significant enrichment (*p* < 1.0 × 10^-16^) in enhancer-associated regions (H3K4me1). Vice versa, these CpGs were significantly depleted in low CpG-context and intergenic regions. Adipose-specific and blood-specific unmethylated CpGs showed very similar genome feature association trends, whereas shared CpGs showed an even stronger enrichment in promoter regions, ranging between 15-fold and 17-fold (*p* < 1.0 × 10^-16^; Additional file 2: Figure S4B).

Genome features of fully methylated CpGs displayed an opposing pattern: invariable CpGs in adipose showed a ~3-fold significant (*p* < 1.0 × 10^-16^) depletion in CGIs, TSS200, and exon1, and 43-fold significant (*p* < 1.0 × 10^-16^) depletion in regions covered by H3K4me3. Additionally, a 2.6-fold depletion (*p* < 1.0 × 10^-16^) was detected in the enhancer-associated H3K4me1 histone mark. Similar trends were observed for methylated adipose-specific, blood, and shared invariable CpGs (Additional file 2: Figure S4C).

When selecting invariable CpGs in a more stringent approach, requiring CpGs to be detected and invariable in five or more, or 10 or more individuals, we observed the same trends as described above with even more pronounced enrichment of unmethylated CpGs in promoter-associated and enhancer-associated regions (Additional file 2: Figures S5 and S6).

### Population differentially methylated CpGs

To identify CpGs displaying differential methylation in a healthy population, we estimated the difference in CpG methylation of overlapping covered CpGs between any two samples (Fisher’s exact test) to methylation levels detected at these CpGs to determine the significance in sequence read distributions. We identified these CpGs as differentially methylated CpGs in population (pDMCs). Using the sequence variant-containing datasets, we detected median differential methylation to be 55 % in adipose and 46 % in blood (Additional file 2: Figure S7A) with extreme differential methylation (>60 % difference) detected at 5.0 % and 3.7 % of total CpGs in adipose and blood, respectively.

**Fig. 3:**
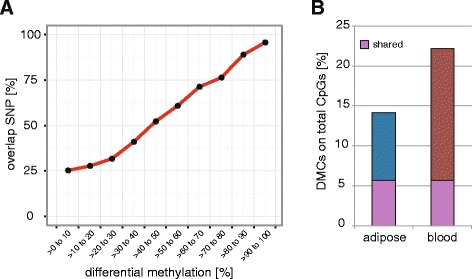
Impact of confounding sequence variants on differential methylation and percentage of population differentially methylated CpGs (*pDMCs*) on total CpGs. **a** Significant DMCs (Fisher’s exact test p < 0.05) determined in filtered blacklisted region-removed but SNP-containing datasets were overlapped with dbSNP137-annoated variants for all samples. Shown is the percentage of DMCs overlapping with annotated SNPs in indicated differential methylation increments. **b** The bar plot displays the percentage of pDMCs on total detected CpGs for adipose and blood in variant-removed datasets. Shared pDMCs are indicated in *purple*

We then overlapped the significant pDMCs with the variant-removed dataset (i.e., dbSNP137-annotated variants excluded) and found that ~25 % of CpGs displaying <10 % differential methylation overlapped with a SNP, this being equivalent to the amount of overall CpGs overlapping a sequence variant (see “Global CpG methylation patterns” above). However, with increasing differential methylation we observed higher confounding variant bias, with pDMCs displaying >90 % of differential methylation being ~95 % confounded by an SNP (Fig. 3a). These findings were shared [31] and highlight the importance of variant filtering for accurate interpretation of differential methylation analyses in unrelated individuals, as we recently showed in public datasets [31]. Removing SNPs among the significant pDMCs reduced the detected extreme differential methylation levels (>60 % difference) to 3.3 % and 1.9 % in adipose and blood, respectively. Concomitant with this we observed a drop in median methylation levels in both tissues (median_Adipose_ = 50 %; median_Blood_ = 41 %; Additional file 2: Figure S7A). Overall, we determined the proportion of pDMCs on total CpGs on autosomes to be 14 % in adipose (40 % of these were shared with blood) and 22 % in blood (26 % of these were shared with adipose) after variant filtering (Fig. 3b). Compared to adipose, we observed lower pDMC methylation levels (Additional file 2: Figure S7B) and a higher percentage of pDMCs on total detected CpGs in blood, which might be explained by the higher cellular heterogeneity of blood, which presumably blunts methylation differences of individual cell populations and increases the number of potentially detectable pDMCs, respectively. Similar differential methylation distribution trends were observed for tissue-specific and shared pDMCs in both tissues (Additional file 2: Figure S7C, D).

We next sought to determine the proportion of significant pDMCs that are due to different cellular heterogeneity across the 27 individuals. We thus correlated methylation levels of each pDMC with proportions of specific blood cell types (i.e., neutrophils, lymphocytes, monocytes, and eosinophils) where at least 10 individuals were covered. We found that 17.5 % of the blood pDMCs were strongly correlated with different proportions of blood cell types (Pearson’s R > 0.5, *p* < 0.05) and this relationship was stronger with increased variation in methylation levels. More specifically, when we restricted the analysis to the top 1,000 pDMCs based on SD, we noted that as much as 24.1 % of the pDMCs correlated with blood cell type proportions (Pearson’s R > 0.5, *p* < 0.05).

We also investigated the genomic features of our significant pDMC (Fisher’s *p* < 0.05; detected in one or more comparison) by overlapping their location with the above introduced genomic regions (Additional file 2: Figure S8): we noted that compared to background (all CpGs detected in two or more individuals) adipose pDMCs were significantly depleted in all CGI-associated and genic regions (Fisher *p* < 1.0 × 10^-16^), and vice versa enriched in low CpG contexts and intergenic regions (*p* < 1.0 × 10^-16^). Strongest depletions were detected in gene promoter regions (3-fold to 5-fold in TSS200, exon 1, H3K4me3; 22.7-fold in CGIs). Blood pDMCs displayed a similar feature association although less pronounced significant depletions (*p* < 1.0 × 10^-16^) were observed in CGI-associated regions, e.g., only 4.5-fold in CGIs. Tissue-specific pDMC feature associations were similar to the ones obtained for the corresponding “complete” adipose or blood, respectively, whereas shared pDMCs associations were similar to the ones observed in adipose (Additional file 2: Figure S8).

### Population differentially methylated regions

Given that our WGBS data were at a relatively low depth at an individual level, we next took advantage of the complete population tissue cohorts to investigate regions of clustered methylation variability, or pDMRs. We determined pDMRs by weighting the variance of CpG methylation across individuals as well as the consistency of CpGs within the region (see “Methods”). For this analysis we considered CpGs covered in three or more individuals, leaving 14.7 × 10^6^ CpGs in adipose and 17.8 × 10^6^ CpGs in blood. We selected the top 10 % of pDMRs covering 23.8 × 10^5^ regions in adipose and 26.2 × 10^5^ regions in blood for further analyses (Additional file 1: Table S5). Of these top pDMRs, we noted that 34–37 % were shared across tissues (Additional file 1: Table S5). Similar to pDMCs, we found that 24.5 % of CpGs mapping to pDMRs were significantly associated with blood cell type proportions (Pearson’s R > 0.5, *p* < 0.05).

Genome feature association results identified pDMRs to be significantly (Fisher’s *p* < 0.05) depleted in gene promoter regions and enriched in enhancer regions irrespective of pDMR category. For instance, we observed adipose pDMRs to be 3.4-fold depleted in CGIs (*p* < 1.0 × 10^-16^), and 3.8-fold depleted in H3K4me3-occupied regions (*p* < 1.0 × 10^-16^) but 3.7-fold enriched in H3K4me1-occupied regions (*p* < 1.0 × 10^-16^) (Additional file 2: Figure S9A). This observation is consistent with previous results showing enhancer regions to be more variable than promoter regions [15, 16, 32]. Overall, pDMC and pDMR genome feature association trends are similar, with the notable exception of adipose pDMCs (within tissue and tissue-specific) not showing enrichment in H3K4me1-occupied regions.

**Fig. 4:**
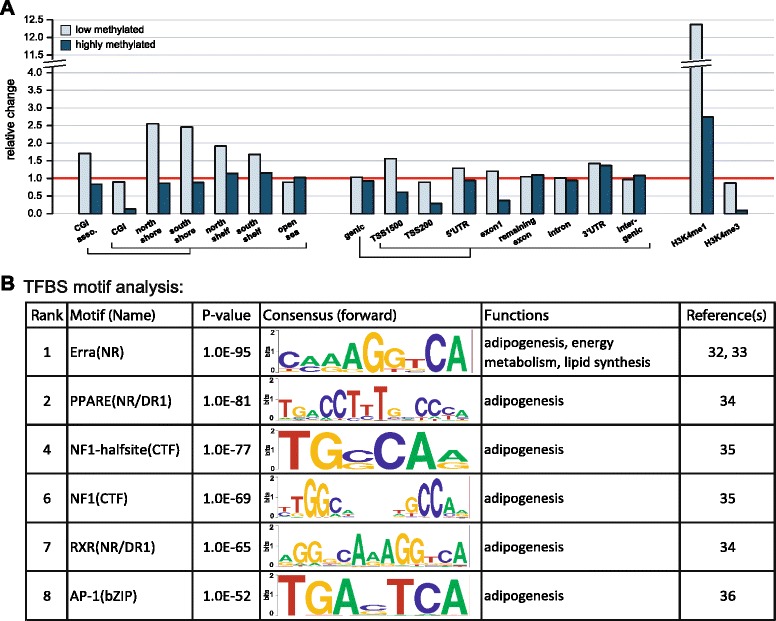
Genomic feature association of adipose-specific population differentially methylated regions (*pDMRs*), and transcription factor binding site (*TFBS*) motif analysis for adipose-specific low-methylated pDMRs. We determined pDMRs using a novel algorithm that weights the variance of CpGs methylation across individuals as well as the consistency of CpGs within the region (see “Methods”). **a** We associated the top 10 % of adipose-specific pDMRs with genomic features. The fold change of pDMR versus background (all CpGs detected in three or more individuals) is shown for each genomic feature. The *red line* demarks the border between enrichment (relative change > 1) and depletion (relative change < 1). **b** TFBS analysis was carried out for adipose-specific low-methylated pDMRs using the Homer software [33]. Shown are selected TFBS motifs including overall rank in the Homer analysis, *p*-value, forward consensus binding sequence, associated function, and references. The complete TFBS motif analysis result is available in Additional file 1: Table S6

We then focused on adipose pDMRs by separating regions based on methylation level: 15 % of pDMRs were identified to be lowly methylated (<50 %) and 85 % of pDMRs were highly methylated (≥50 %). Compared to all CpGs detected in three or more individuals, low-pDMRs were 12.5-fold enriched in enhancer-associated H3K4me1 regions (*p* < 1.0 × 10^-16^); high-pDMRs displayed a 10.9-fold depletion in gene promoter-associated H3K4me3-occupied regions (*p* < 1.0 × 10^-16^) and, compared to low-pDMRs, only slight but significant enrichment in the enhancer-associated H3K4me1-occupied regions (*p* = 3.7 × 10^-9^) was noted (Fig. 4a). Observed low-pDMRs displayed strong enrichment in enhancer regions; to identify TFs binding in these potentially open chromatin regions and associated functions, we carried out a TF binding site (TFBS) motif analysis within these low-pDMRs mapping to the enhancer regions against all remaining low-pDMRs (i.e., those not overlapping such regulatory element) [33]. We found that the binding motif of estrogen-related receptor alpha (*ERRA*)—a TF known to be involved in adipogenesis, energy metabolism, and lipid synthesis [34, 35]—to be the most significantly (*p* = 1.0 × 10^-97^) enriched TFBS in these regions. Additional significantly enriched motifs included TFBSs for *peroxisome proliferator-activated receptor γ* (*PPARG*; *p* = 1.0 × 10^-84^), *retinoid X recepto*r (*p* = 1.0 × 10^-65^), *nuclear factor 1/CAAT-binding transcription factor* (*p* = 1.0 × 10^-78^ and *p* = 1.0 × 10^-67^), and *activator protein 1* (*p* = 1.0 × 10^-53^), which are all TFs known to be involved in adipocyte differentiation [36–38] (Fig. 4b, Additional file 1: Table S6).

Finally, focusing on adipose high-pDMRs we carried out a pathway analysis (Ingenuity) for regions close to the TSS of genes and identified lipid metabolism as the most significantly associated function (*p* = 1.5 × 10^-5^). In addition to this stringent threshold, we also identified pDMRs with less stringent thresholds, selecting the top 20 % and 25 % of CpGs per tissue (Additional file 1: Table S5), and observed genome feature associations similar but less pronounced to the ones for stringently (top 10 %) selected pDMRs (Additional file 2: Figure S9B-E).

### Differentially methylated CpGs of genetic versus environmental origin

Inter-individual methylation variation can be attributed to genetic and non-genetic effects. Non-genetic effects can be further divided into familial (i.e., shared environmental) and non-familiar (i.e., unique/non-shared environmental or stochastic) effects. Twin studies provide the ideal model to calculate the proportion of methylation variation attributable to these different factors. Here, we calculated intra-class correlations (ICC) of the identified adipose pDMCs that were covered in at least five MZ and five DZ adipose samples (N = 4,798) to estimate additive genetic effects, shared environmental effects, and non-shared environmental effects. We found that for 37 % of the pDMCs, additive genetic effects accounted for >30 % of the total variance in methylation. In contrast, shared environment seem to have a minor role; only 3 % of the pDMCs had shared environment contributing >30 % to adipose methylation variation, indicating that the remaining proportion of the non-genetic variance was due to non-shared environment and/or stochastic factors. In fact, >60 % of the pDMCs were estimated to have non-shared environment accounting for >90 % of the variance. Interestingly, these environmentally driven pDMCs were shown to be depleted in promoters (*p* = 2.4 × 10^-13^) but enriched in coding regions (*p* = 3.9 × 10^-4^) when compared to all pDMCs detected in adipose tissue (Additional file 2: Figure S10).

We sought to characterize these abundant non-shared environmental DMCs in more detail; for simplicity, we refer to these CpGs as “environmental” in origin or eDMC. For this purpose we estimated DMCs within two MZ pairs per tissue sequenced at moderate coverage (MZ2, MZ3; ≥10-fold in adipose, ≥19-fold in blood; Additional file 1: Table S1) and compared these with a similarly powered analysis in unrelated individuals. First, at *p* < 0.05 (Fisher’s exact test) we identified on average 1.13-times (adipose) and 1.3-times (blood) more DMCs in unrelated samples versus MZ twins, confirming genetic effects on CpG methylation variation when overlapped SNPs are accounted for. Second, as shown in the heritability analysis, a large proportion of DMCs seemed to be of non-shared environmental origin (i.e., eDMCs) and we estimated non-shared environmental effects to account for over 75 % of methylation variation in blood (Additional file 2: Figure S11A). To study this pattern further we analyzed the identical set of blood samples (MZ2 and MZ3) using a targeted capture-based approach for bisulfite sequencing of functional elements, MCC-seq, that we recently introduced [27]. Carrying out the same analysis in this independent higher coverage dataset (MZ2: >22-fold; MZ3: >25-fold) we estimated eDMCs to account for 64 % of all detected DMCs. This lower proportion of eDMCs in targeted MCC-seq versus WGBS was in fact not due to differential coverage but rather due to the predominance of enhancer/promoter regions targeted in the former method. More specifically, when we filtered the WGBS-derived DMCs to only cover overlapping MCC-seq functional elements, we found the same proportion (65 %) of total DMCs (Additional file 2: Figure S11A). These findings are in agreement with genome feature annotation of purely environmentally driven adipose pDMCs from the twin analysis, showing genic regions to be influenced to a larger extent by unique environmental or stochastic factors (Additional file 2: Figure S11B).

### Functional characterization of differentially methylated CpGs of non-shared environmental origin

**Fig. 5:**
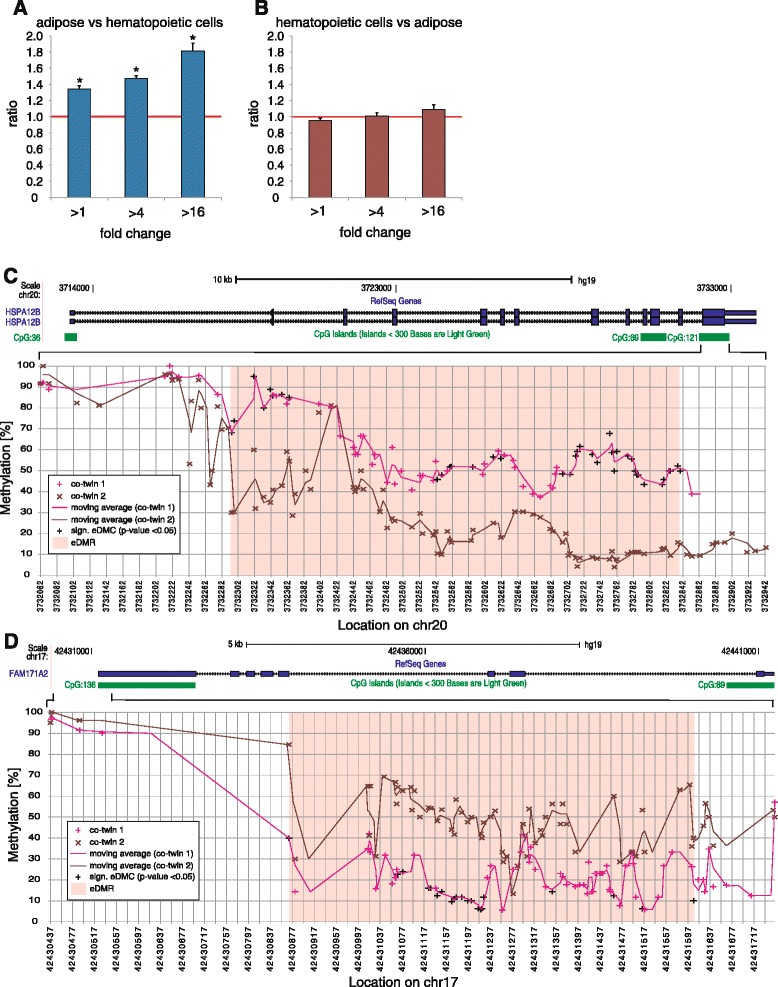
Characterization of differentially methylated CpGs of non-shared environmental origin (*eDMR*). **a** eDMRs identified in adipose were associated with genes by overlapping with RefSeq-annotated genes (defined as genic regions including 10 kb downstream of the transcription start site); DeSeq normalized total stranded RNA-seq data were used to determine fold expression changes for RefSeq genes between subcutaneous adipose tissue and T-cells, monocytes, and B-cells. The number of genes significantly (DeSeq-adjusted *p* < 0.05) overexpressed in adipose versus each hematopoietic cell type (>1-fold, >4-fold, >16-fold higher expression in adipose) was determined for eDMR-associated and all RefSeq genes; the latter to identify dynamic adipose-specific genes. Shown is the ratio of eDMR-associated versus RefSeq genes averaged across the three hematopoietic cell types corrected for background at indicated fold overexpression levels in adipose. *Stars* indicate z-score-derived *p*-values <0.001. **b** Same as (a) but comparing eDMR-associated genes overexpressed in hematopoietic cells to adipose. **c** (*top*) UCSC-derived scheme of the *HSPA12B* RefSeq gene locus and CGIs in the gene region (hg19). (*bottom*) Zoom into *HSPA12B* gene region overlapping CGI CpG: 121. For each twin within MZ2, methylation levels are shown for each detected CpG site. The trendl ine was determined using a moving average (period = 2). Methylation values highlighted in *black* in co-twin 1 indicate significant eDMCs (Fisher’s exact test *p* < 0.05), the associated eDMR is highlighted in *rose*. **d** (*top*) UCSC-derived scheme of the *FAM171A2* RefSeq gene locus and CGIs in the gene region (hg19). (*bottom*) Zoom into *FAM171A2* gene region overlapping CGI CpG: 136. Illustration as in (**c**)

We next investigated whether eDMCs cluster in regions and can be linked to potential functional relevance. We screened 500 bp regions upstream and downstream of each eDMC identified at nominal significance (*p* < 0.05), and defined regions containing three or more eDMCs displaying unidirectional differential methylation as eDMRs, with overlapping eDMRs being merged. In adipose, we identified 54 and 75 eDMRs, comprising 0.27 % and 0.32 % of eDMCs in the MZ2 and MZ3 pairs, respectively (false discovery rate [FDR] ≤ 0.1 % based on permutation test). In blood, we observed 923 and 386 eDMRs, comprising 1.66 % and 0.82 % of eDMCs in the MZ2 and MZ3 pairs, respectively (FDR ≤ 0.1 % based on permutation test). All identified eDMRs were tissue-specific and not shared among MZ pairs. We then sought to test for potential tissue-specific regulation of eDMR-associated genes and thus overlapped eDMRs with RefSeq-annotated genes. RNA-seq-derived gene expression data were available for subcutaneous adipocytes as well as for normal primary B-cells, T-cells, and monocytes (all samples from an unrelated study cohort) [27]. First, we compared the expression level for eDMR-associated genes (eDMRs were mapped to genic regions including 10 kb downstream of the TSS) between adipocytes and hematopoietic cell types. eDMR-associated genes were significantly enriched in genes with higher expression in adipose tissue than in blood, with enrichment increasing from 1.34-fold for genes solely over-expressed (z-score derived *p* < 0.001) in adipose tissue to 1.8-fold in genes >16-fold over-expressed (*p* < 0.001) in adipose (Fig. 5a), suggesting that adipose eDMRs map to dynamic genes specific to adipose tissue. For blood-associated eDMRs we did not observe similar enrichment in genes with elevated expression in hematopoietic cells (Fig. 5b). However, pathway analysis of blood eDMR-associated genes identified hematological system development and function (*p* = 1.1 × 10^-3^) as well as humoral immune response (*p* = 1.1 × 10^-3^) as the top associated bio functions.

These findings indicate that inter-twin eDMR count differences may arise from differences in blood heterogeneity. To address this we performed a similar analysis as previously, correlating pDMC methylation levels of each CpG in the defined eDMRs with proportions of specific blood cell types (i.e., neutrophils, lymphocytes, monocytes, and eosinophils) where at least 10 individuals were covered. We found that eDMCs were to a lesser extent confounded by different cell heterogeneity than all DMCs in the population (12.6 % versus 24.5 %). Because we observed a striking difference in the number of blood eDMRs between the two twin pairs (i.e., N_eDMR_MZ2_ = 923 versus N_eDMR_MZ3_ = 386), we also compared the confounding effect of cell type across the two pairs. We found only a slightly higher number of CpGs impacted by cell type proportions in MZ2 than in MZ3 (13.3 % versus 11.9 %, *p* = 0.42), so the difference in eDMRs between the two twin pairs seem to be only to a minor extent affected by differences in cell counts. The difference may rather be explained by different environmental exposure impacting CpG methylation, such as tobacco smoking, which represents the most commonly studied and proven environmental factor impacting methylation [39–42]. Indeed, both MZ2 siblings have been smoking for the past 40 years, whereas the MZ3 siblings had not consumed cigarettes within the last 20 years. To further investigate the direct impact of smoking on the blood methylome in our study cohort, we compared MZ2 and MZ3 DMCs to replicate array-derived loci differentially methylated in smokers versus never-smokers [41, 42], based on the Zeilinger et al. report that methylation levels of former smokers regain levels observed in never smokers [41]. We observe significant (Fisher’s p < 0.05) enrichment of significant DMCs overlapping known smoking loci compared to all jointly detected CpGs for three out of four comparisons, and all observed smoking-associated DMC methylation trends match previously reported ones (Additional file 2: Table S7). Interestingly, within MZ2 siblings we identified a blood eDMR comprising 37 significant eDMCs within a CpG island overlapping the *HSPA12B* locus (Fig. 5c) reported to be involved in asthma with interaction of environmental tobacco smoke [43].

Finally, further investigation of blood eDMRs identified one region comprising 21 eDMCs overlapping a CpG island in *FAM171A2* (Fig. 5d). Interestingly, *FAM171A2* was linked to platelet count in a recent genome-wide association study [44]; comparing with all available MZs, we in fact observed a differential platelet count of MZ2 to be in the highest third. Because this region was not covered in MZ3, this finding can only be considered an indication that *FAM171A2* differential methylation is associated with platelet count.

### Inter-tissue epigenetic drift

Variation in CpG methylation within a tissue over time is often referred to as aging epigenetic drift. Similarly, methylation variation across tissues could then be considered inter-tissue epigenetic drift. We recently showed that a large proportion of CpGs associated with common genetic variants are stable across tissues [14]. This, together with our finding that the majority of pDMC are purely of non-shared environmental origin, may indicate that genetic factors have the ability to limit inter-tissue epigenetic drift. To test this hypothesis, we first focused on DMCs that were identified to be shared across tissues (i.e., adipose and blood). We again utilized the two MZ pairs per tissue sequenced at moderate coverage (MZ2 and MZ3). We selected all tissue-shared pDMCs (Fisher’s *p* < 0.05) covered in all four adipose MZ2 and MZ3 samples (N = 155,172). We then enriched this set of tissue-shared pDMCs to reflect those impacted by genetic variation by selecting the top 50 % (N = 77,586) of CpGs displaying th highest “across twin adipose methylation variance/within twin adipose methylation variance” ratio. We further restricted our analysis to CpGs also covered in the four blood MZ2 and MZ3 samples and excluded adipose and blood eDMCs, resulting in 31,167 CpGs. Reads for these CpGs were then merged within each pair and tissue, followed by an MZ2 versus MZ3 pairwise comparison that revealed 378 tissue-shared DMCs (Fisher’s *p* < 0.05). Of note, 70 % (N = 265) of these CpGs showed the same direction of effect across tissues, which is a significant deviation from the expected distribution under the null hypothesis (uniform random distribution expected; exact binomial *p* = 3.4 × 10^-15^) and most likely reflects genetic effects. We then undertook a similar approach for eDMCs and selected significant eDMCs (Fisher’s *p* < 0.05) that were shared across tissue (MZ2: N = 1,098; MZ3: N = 1,228) and then merged the reads across tissues. We performed new pairwise analyses within each pair and noted that only ~50 % of the eDMCs remained significant per pair (MZ2: N = 555; MZ3: N = 640); thus there was no significant deviation from expected effects under the null hypothesis (uniform random distribution expected; MZ2 exact binomial test *p* = 0.74, MZ3 *p* = 0.15).

Second, we used the ICC estimates of the 4,798 adipose pDMC included in the analysis of the estimation of additive genetic, shared, and non-shared environmental effects but divided those into adipose-specific and tissue-shared. We then compared the heritability and non-shared environmental proportions across the two groups. We found the proportions of heritable CpGs to be significantly larger in the tissue-shared group than in the adipose-specific group (Fisher’s *p* = 0.004-0.04, Additional file 1: Table S8). In contrast, purely environmentally driven pDMCs were more tissue-specific (Fisher’s *p* = 0.02, Additional file 1: Table S8).

Taken together, genetic variation seems to significantly constrain inter-tissue epigenetic drift as opposed to non-shared environmental factors, which impact the methylome in a tissue-specific manner.

### The non-CpG methylation landscape

Earlier genome-wide investigations employing multiple tissues but few replicates per tissue have identified that non-CpG and CpH methylation comprise up to 25 % of all cytosine methylation in embryonic stem cells [2] and induce pluripotent cells [45], decreases upon differentiation, and are near absent in investigated differentiated cells and tissues including whole blood [2, 45], with exceptional somatic CpH methylation observed in brain tissue and placenta [46]. Notably, the genome-wide CpH methylation in adipose tissue is unknown. Consequently, we investigated CpH methylation from our adipose and blood WGBS effort. We restricted our analysis to sites covered four or more reads applying SNP/blacklist filtering (removal of 4.2 % of Cs). In total we detected 9.63 × 10^8^ and 1.00 × 10^9^ CpHs in adipose and blood, respectively, with even distribution of CpH coverage on the forward and reverse strands (Additional file 1: Table S9). CpH methylation was then determined by applying a stringent detection cut-off of >50 % methylation per CpH in two or more individuals to exclude random bisulfite conversion artifacts biasing our analysis. Comparing to CpG methylation of >50 % (in the filtered and variant removed datasets) for each individual, we determined CpH methylation to comprise on average 9.3 % (SD = 2.2 %) and 8.6 % (SD = 3.2 %) of total cytosine methylation in adipose and blood, respectively. Across the population we identified CpH methylation to impact in total 1.7 × 10^6^ (0.18 % of all CpHs) and 2.3 × 10^6^ (0.23 %) CpHs in adipose and blood, respectively. We observed a dinucleotide methylation frequency of CpA > CpT > CpC—a trend that is consistent with previous WGBS studies [2, 3, 18, 19], and roughly one third CpHpG and two thirds CpHpH methylation, with even overall strand distribution (Additional file 2: Figure S12A). Genome feature association of CpH methylation compared to CpH background (all CpH detected in two or more individuals) revealed in adipose and blood a 1.6-fold to 2.9-fold depletion (Fisher’s *p* < 1.0 × 10^-16^) in regions displaying no or low CpG methylation (CGI, TSS200, H3K4me3, H3K4me1) (Additional file 2: Figure S12B). This indicates that CpH methylation in these two tissues occurs mainly in the presence of CpG methylation, and might occur by potentially “erroneous/unspecific” methylation through the CpG methylation machinery.

To study this in more detail we investigated the relationship between CpH methylation and methylation of nearby CpGs and found for 95 % of methylated CpHs in adipose and 97 % in blood that the closest CpGs displayed ≥50 % methylation, with an average distance between CpH and CpG of ~150 bp. When restricted to the closest detected CpG within 10 bp only, these numbers increased to 99.7 % in adipose and 99.6 % in blood, underlining the possibility of potential concomitant CpH methylation in close proximity to CpG methylation. Next, we sought to characterize the remaining 5.2 % and 3.2 % of CpHs in adipose and blood, respectively, that were ≥50 % methylated but in proximity to CpGs displaying <50 % methylation (highCpH-lowCpG). We found that 40 % of adipose and 59 % blood highCpH-lowCpG methylation was shared between tissues. To investigate whether highCpH-lowCpG methylation is clustered, we screened for concomitant methylation within 500 bp around each site within individuals. Considering ≥3 CpHs as a cluster we detected 62 % of these sites to be clustered per tissue. As expected, genomic feature association identified adipose and blood highCpH-lowCpG methylation as enriched in promoter-associated and enhancer-associated regions (Fisher’s *p* < 1.0 × 10^-16^, Additional file 2: Figure S12C). However, in both tissues we also observe as >2.5-fold enrichment in CGI shores (Fisher’s *p* < 1.0 × 10^-16^, Additional file 2: Figure S12C), suggesting that highCpH-lowCpG methylation might be observed in areas of CpG methylation transition, i.e., be located between unmethylated and methylated CpGs and be detected as highCpH-lowCpG as it is in closer proximity to unmethylated CpGs.

## Discussion

By generating full genome single-base resolution methylation maps of 34 adipose and 27 whole blood samples employing WGBS, we report the first comprehensive and unbiased analysis of human CpG and CpH methylation variation at the population level. We identified sequence variants at CpGs to underlie the majority of extreme differential methylation, and when taking this into account we estimate that ~15–20 % of CpGs are differentially methylated in the population. Our estimate of the dynamic population methylome of a tissue is in line with earlier studies investigating inter-tissue methylome variation [15–17]. We also note that, similar to these cross-tissue epigenome-mapping efforts, population-based methylome analysis at single-site resolution can be used to map potentially regulatory elements. More specifically, we show how dynamic regions map preferentially to distal-regulatory elements whereas CpGs that are invariable in a population are mainly located in promoter-associated regions.

Our study design with mean genome coverage of ~6–9-fold mirrors recent suggestions by the NIH Roadmap for coverage requirements for the detection of functionally relevant methylation changes [21]; however, leaving minor differences in methylation levels across individuals (i.e., <20 %) unidentifiable as the detection of such small differences in methylation would require ~30–50-fold sequence coverage. Sequencing to the latter depth is still cost-prohibitive for large population studies and, given the relative small proportion of the methylome being variable across tissues or across individuals in a given tissue rather than by WGBS, population methylation variation might be interrogated by more cost-efficient targeted approaches once a tissue-specific methylome variability map is established. Indeed, we recently implemented a methylome capture approach based on dynamic sites in adipose identified in this study (in combination with other regions) that allows highly efficient and comprehensive interrogation of adipose methylome variability [27]. However, we also report that all differentially methylated CpGs in the population are enriched in intergenic regions and low CpG content regions, as opposed to CpGs that are variable due to a non-shared environment which are enriched in genic regions. Thus, when targeting population-variable “functional sites,” the full scope of methylation variation remains underappreciated.

We also highlight how population-based low pass WGBS can be used for powerful discoveries of clustered regions of differentially methylated CpGs by weighting within-population variability as well as the within-region consistency. Applying this new algorithm we identified population-variable clusters of methylation sites (pDMRs). Whereas previous approaches investigating tissue-specific DMRs employed Hidden Markov models, determining variance across the population followed by segmentation and selection for highly variable regions [16], or applied a random effects model incorporating methylation variability information derived from pairwise comparisons [15], our approach additionally incorporates information about the consistency of a DMR profile across the population. Because we were dealing with populations, we assumed that profile inconsistencies would potentially yield false positive results, hence excluded these. Functional annotation of these pDMR seemed to reflect tissue-specific signatures and again highlights that extension of epigenome mapping across population samples may provide new insights into regulatory regions, and indeed may be more useful in the context of phenotypic correlations than cross-tissue maps because static “tissue-indicators” are excluded. For instance, dynamic regions in adipose were mapped to genes enriched in lipid metabolism pathways, and to enhancer elements harboring binding site motifs for TFs involved in adipocyte differentiation, energy metabolism, and lipid synthesis. Interestingly, the most significantly enriched TFBS was the motif of *PPARγ*, a master regulator of adipogenesis with critical involvement in insulin sensitivity. Recently, *PPARγ* activation by the insulin sensitizer (antidiabetic) drug rosiglitazone was shown to correlate with enhancer RNA (eRNA) expression almost exclusively at *PPARγ*-binding sites, inducing the recruitment of transcriptional co-activators [47]. In contrast, transcriptional repression was concomitant with eRNA downregulation at sites devoid of *PPARγ* but enriched in other TFs [47]. Our data show population-variable epigenetic signatures in regulatory mechanism in *PPARγ* enhancer regions, with potential impact on inter-individual response to treatments.

Our study design included not only population-based samples but also family structure with both MZ and DZ twins. This allowed us to disentangle genetic and non-genetic effects (shared and non-shared environment) on the population methylome. We estimated that >60 % of DMCs are purely of non-shared environmental origin (referred to as eDMCs), and shared environment seems to play only a minor role in shaping the dynamic methylome. We highlight that these eDMCs are significantly clustered in regions and thus likely of true environmental origin rather than biological noise in tissue development. In fact, by incorporating a differential blood count, we show that clustered regions of eDMCs are to a lesser extent cofounded by blood cellular heterogeneity than the complete set of population DMCs. One of these non-shared environment regions allowed us to make links between methylation signatures in blood and tobacco smoking, thus potentially reflecting the wide impact this specific exposure has on multiple circulating cell types. We also identified a potential link between pronounced methylome variation and platelet count because one of our eDMRs mapped to *FAM171A2*, which has been associated with platelet count in a large genome-wide association study [44]. This could be an example where both environment and genetic variants work in concert. We note though that while platelets have no nuclear genome, their variation may be correlated with other blood counts, raising the possibility of a spurious association. This highlights the importance of applying accurate statistical models that adjust for cellular composition in epigenome-wide association studies of disease traits when peripheral blood is used as the discovery tissue.

We also applied the twin structure to study how genetic and environmental factors impact inter-tissue epigenetic drift, utilizing the dynamic CpGs shared across both tissues. Here we found that only a minority of CpG genetic variants had a significant effect in limiting inter-tissue drift. More strikingly, effects of environmental origin completely lack such ability, exerting their effect only in a tissue-specific manner. Thus, as only a minority of methylation changes seems to be stable across tissues, it is of great importance to conduct epigenome-wide association studies in tissues closely related to the trait of interest because otherwise a major fraction of association might not be captured.

Finally, we also investigated population CpH methylation in both tissues, identifying 94.8 % of methylated CpHs in adipose and 96.8 % in blood to be in close proximity to a methylated CpG. This is in line with a previous study employing reduced representation bisulfite sequencing to investigate CpA methylation across tissues, which identified that CpH methylation is spatially correlated with CpG methylation [45]. These observations hint towards potential CpG methylation machinery errors facilitating CpH methylation. Closer investigation of remaining highCpH-lowCpG methylation identified enrichment in areas of methylation transition. We did not observe a clearly preferred cytosine sequence context or an indication of genetic influence on highCpH-lowCpG methylation. Overall, our observations do not support (and also do not conclusively exclude) the functional relevance of CpH methylation in investigated tissues.

## Conclusions

Our study highlights the utility of low pass population WGBS in identifying differentially methylated sites and regions. Non-shared or unique environmental factors that act mainly in a tissue-dependent manner were shown to be the main source of human methylome variation. We identified that methylation variation occurs mainly outside promoter regions, thus demonstrating the limitation of current array-based population methylation studies and underlining the importance of expanding these studies beyond promoter regions, specifically into enhancer regions. The presented results may also function as a guide to design targeted panels for cost-effective and comprehensive evaluation of only variable methylation in investigated tissues. However, we note that when targeting the known “functional” methylome, the full scope of environmental variation remains underappreciated.

## Methods

### Study cohort

Participating in this study were 43 Caucasian female twins belonging to the TwinsUK/MuTHER cohort [23, 48]. Primary subcutaneous adipose tissue was collected from 34 individuals, and whole blood samples from 27 individuals, as described previously [48]. In brief, skin punch biopsies were taken from a relatively photo-protected lower abdominal site, and adipose tissue biopsies were dissected from the same incision. Peripheral blood samples were taken simultaneously, and all samples were stored immediately in liquid nitrogen [48].

### Ethics

All sample collection procedures followed were in accordance with the ethical standards of the St. Thomas’ Research Ethics Committee (REC reference 07/H0802/84) at St. Thomas’ Hospital in London, and all study participants provided written informed consent. The study has also received certification of ethical acceptability for research involving human subjects by the Faculty of Medicine Institutional Review Board at McGill University (IRB Assurance number: FWA00004545). Finally, all experimental procedures complied with the Helsinki Declaration.

### DNA extraction

Genomic DNA (gDNA) was isolated using the NORGEN purification kit (Norgen Biotek Corporation, Thorold, ON, Canada) according to manufacturer’s protocol. All quantifications were carried out using Quant-iT PicoGreen (Life Technologies, Burlington, ON, Canada).

### Array-based methylation profiling

Using the HumanMethylation450 BeadChip (Illumina, San Diego, CA, USA), methylation profiles of all adipose tissue samples have previously been generated [14]. In short, 700 ng gDNA per sample were bisulfite converted using the EZ-96 DNA Methylation kit (Zymo Research, Irvine, CA, USA) according to manufacturer’s protocol. Next, 5 μL of bisulfite-converted DNA were processed and arrayed on HumanMethylation450 BeadChips as described by Illumina. For the remaining samples, methylated (M) and unmethylated (UM) signal intensities were quantile normalized for each probe type separately and β-values were calculated using R as (Mnormalized)/(UMnormalized + Mnormalized), with β ranging from 0 (no methylation at any allele) to 1 (full methylation at both alleles) [14]. The BeadChip contains a total of 485,577 probes, of which we discarded probes with ≥90 % sequence similarity to multiple genomic locations, probes with sequence variants in the probe binding region (1000 Genomes Phase I integrated variant set release (v3); probes containing two or more SNPs), and probes located on the Y chromosome, leaving a total of 385,553 probes for further analyses.

### Whole genome shotgun bisulfite sequencing

WGBS gDNA library preparations were carried out using the TruSeq DNA Sample Prep Kit v2 (Illumina) with an added bisulfite conversion step. Briefly, 1–3 μg of gDNA was fragmented to 300–400 bp peak size using the focused-ultrasonicator E210 (Covaris, Woburn, MA, USA) to generate double-stranded DNA with 3′ or 5′ overhangs. Fragment size distribution was controlled on a Bioanalyzer DNA 1000 Chip (Agilent, Mississauga, ON, Canada). End repair, sample purification with AMPure beads (Beckman Coulter, Mississauga, ON, Canada), adenylation of 3′ ends, and adaptor ligation was carried out as per Illumina’s recommendations. The ligation product was cleaned-up by one AMPure purification step, the purified DNA was then analyzed on a Bioanalyzer High Sensitivity DNA Chip (Agilent), and quantified by PicoGreen before undergoing bisulfite conversion using the Epitect Fast DNA Bisulfite Kit (Qiagen, Toronto, ON, Canada) according to manufacturer’s protocol. Bisulfite-converted DNA was quantified using OliGreen (Life Technologies), and based on quantity amplified by four to six cycles of PCR using the Hifi Uracil + DNA polymerase (Kapa Biosystems, Woburn, MA, USA) according to manufacturer’s protocol. Amplified libraries were validated and quantified on Bioanalyzer High Sensitivity DNA Chips and underwent 100 bp paired-end sequencing on Illumina HiSeq2000 or HiSeq2500 systems. Generated sequencing reads were aligned to the bisulfite-converted reference genome using Burrows–Wheeler alignment: (i) clonal reads, (ii) reads with low mapping quality score (<20), (iii) reads with >2 % mismatch to converted reference over the alignment length, (iv) reads mapping on the forward and reverse strand of the bisulfite-converted genome, (v) read pairs not mapped at the expected distance based on library insert size, and (vi) read pairs that mapped in the wrong direction were removed as described by Johnson et al. [49]. To exclude sample mix-ups we determined variants using Bis-SNP [50] and compared them to available microarray or sequencing-derived genotype information of the same samples. Further, we overlapped loci highly covered in our WGBS data with DAC Blacklisted Regions (DBRs) and Duke Excluded Regions (DERs) generated for the ENCODE project (http://hgwdev.cse.ucsc.edu/cgi-bin/hgFileUi?db=hg19&g=wgEncodeMapability) [24]. Both DBRs and DERs comprise blacklisted regions in the human genome that yield artifactually high read counts across tissues and cell lines in next-generation sequencing and were defined based on DNaseI, FAIRE, and ChIP sequencing experiments. Overall, the tendency of CpGs to overlap with blacklisted regions increased irrespective of tissue with (i) increasing coverage, i.e., all loci covered more than their sample-specific coverage plus 50-fold the SD of coverage in at least one individual overlap blacklisted regions; and (ii) increasing number of individuals in whom loci are detected as highly covered, i.e., loci, which were detected in five individuals being covered more than average individual-specific coverage plus 3–4-fold the SD of coverage had a >60 % probability to overlap blacklisted regions (Additional file 2: Figure S13). To exclude these potentially artifactual regions from downstream analyses we removed DBR and DER blacklisted regions, covering in total 19,048,164 bp on autosomes and chromosome X. Additionally, we determined a study-specific threshold excluding loci covered more than the sample-specific coverage plus 1.5-fold the SD of coverage in at least two samples, thereby removing 17,893 loci from further analysis. To remove confounding sequence variants we filtered for SNPs annotated in dbSNP137 [25].

### RNA sequencing

RNA sequencing has been described previously [27]. In short, subcutaneous AT was available for four obese individuals undergoing bariatric surgery, and B cells, T cells, and monocytes were available from four healthy individuals (Uppsala Blood Transfusion Center, Uppsala University Hospital, Sweden). RNA was isolated from adipocyte cells extracted from AT [51, 52] and hematopoietic cells using the miRNeasy Mini Kit (Qiagen) according to manufacturer’s protocol. We used as input 500 ng RNA (RNA integrity number > 7) for library preparations using the Illumina TruSeq Stranded Total RNA Sample preparation kit according to manufacturer's protocol. Final libraries were quality controlled on a Bioanalyzer and underwent 100 bp paired-end sequencing on the Illumina HiSeq2000 system. Generated raw reads were filtered for quality (phred33 ≥ 30) and length (n ≥ 32), and adapter sequences were removed using Trimmomatic v. 0.32 [53]. Reads passing filters were then aligned to the human reference (hg19) using TopHat v.2.0.10 [54] and bowtie v.2.1.0 [55]. UCSC gene counts were obtained using htseq-count v.0.6.1 (http://www-huber.embl.de/users/anders/HTSeq), and differential expression analysis was carried out using DESeq [56].

### Identification of unmethylated and low-methylated regions

Filtered variant-free methylation data for all individuals were merged for each tissue, keeping only sites detected in three or more individuals (equals ≥12-fold coverage per site). Low-methylated regions and unmethylated regions were identified using the R/Bioconductor package MethylSeekR [26]. Briefly, a cutoff method was utilized wherein unmethylated and low-methylated regions were predicted at single-base resolution as regions of consecutive CpGs with methylation statuses under a set level. We applied default settings (methylation threshold of 50 % and FDR of 5 %). PMD filtering was not required because the alpha distribution was polarized.

### Region definitions and genomic feature association

NCBI reference sequence gene and CGI annotations (hg19) were downloaded from UCSC on 10 September 2013. Regions were defined as (i) CGI-associated regions to be located in a CGI, a CGI shore 2 kb upstream (north) or 2 kb downstream (south) of a CGI, a CGI shelf 2 kb upstream (north) or 2 kb downstream (south) of a CGI shore, or—if in none of the above—to be located in open sea (Additional file 2: Figure S14A); (ii) gene-associated regions to be located 201–1500 bp upstream of the TSS (TSS1500), 200 bp upstream of the TSS (TSS200), in the 5′UTR, exon 1, any of the remaining exonic regions, intronic regions, 3′UTR, or—if in none of above—to be located in intergenic regions (Additional file 2: Figure S14B); or (iii) H3K4me3 and H3K4me1 overlapping regions derived from ChIP-seq data generated by the NIH Roadmap Epigenomics Project [28]. ChIP-seq data analysis was carried out as described previously [14]. In short, aligned H3K4me1, H3K4me3, and input ChIP-seq reads (.BAM files) generated for adipose tissue from five independent donors to the NIH Roadmap Epigenomics Project were downloaded from the Gene Expression Omnibus repository accession numbers [GSM621425, GSM669908, GSM669975, GSM670045, and GSM772757] for H3K4me1; [GSM621435, GSM669925, GSM669988, GSM669998, and GSM670041] for H3K4me3; and [GSM621401, GSM669934, GSM669940, GSM669984, and GSM670043] for the ChIP-seq input files. For data processing, H3K4me1 and H3K4me3 data as well as the input data were divided into 100 bp bins, and the number of reads within each bin was counted. Normalized signals intensities were generated by normalizing the counts per bin to total number of reads, and normalized signal intensities of input were subtracted from ChIP-seq bin data. Data for each mark were then ranked according to normalized signal intensities, and the top 1 % (or other threshold as indicated in text) bins per mark were kept for further analyses. Top-ranked bins were further filtered, keeping only those that were present in at least three individuals for either mark. In genome feature associations, only H3K4me3 mark bins within 1 kb of the TSS of known RefSeq transcripts were considered for promoter mapping, and enhancers were identified using the top 1 % H3K4me1 mark, discarding all data additionally overlapping the H3K4me3 mark. Genomic feature associations were carried out in bedtools [57] and R [58], applying standard functions and the “GenomicRanges” package.

### Population differentially methylated regions

We determined pDMRs by weighting the variance of CpG methylation across individuals as well as consistency of CpGs within the region (Additional file 2: Figure S15). We removed CpGs covered by fewer than three individuals and calculated SD values for the remaining CpGs across the population. We then scanned the methylomes with a sliding window of 500 bp length, discarding regions with single CpGs as well as regions that were subsets of bigger regions. We further calculated the average SD values and the consistency scores for each CpG region. The consistency score for each CpG region was defined as follows:

$$ consistency=\frac{1}{m}{\displaystyle \sum_{i=1}^m}{r}_j, $$

where m is the number of individuals covering this region, is the Spearman correlation between the methylation profile of each individual (i) and the average methylation profile across all individuals of the considered CpG region. Final pDMRs were selected to include the top 10 % (top 20 % or top 25 %) of the above calculated CpG regions, filtered for regions with fewer than three CpGs, and the putative intervals merged into non-overlapping regions (with zero-base-pair-merge).

### Statistical data analysis

Data analyses were carried out in R [58] applying standard functions and the “GenomicRanges” package. ICC values for pDMCs were calculated using the R package irr (v0.84) and only ICCs obtained for both MZ and DZ pairs were kept. Twin-based DNA methylation heritabilities (additive genetic effect) were estimated as twice the difference of the ICC between MZ and DZ twins. Shared environmental effects were estimated by subtracting the heritability estimate from the MZ ICC.

### Transcription factor binding site motif analysis

TFBS motif analysis was carried out using the Homer software [33] applying default settings. Adipose-specific low-pDMRs overlapping the top 1 % of binned ranked H3K4me1 data (see above) were used as input, adipose low-pDMRs not overlapping the top 10 % of binned ranked H3K4me1 data were used as background (4,266 regions in total). TFBS motif consensus sequences were generated using the STAMP software [59] applying default settings; only forward consensus sequences are shown.

### Ingenuity pathway analysis

We performed core analyses considering direct and indirect relationships, including endogenous chemicals, and set thresholds to a maximum of 35 molecules per network and 25 networks per analysis. We included all available data sources, selected for the human species, tissue and primary cells, and experimentally observed molecules and/or relationships only, and selected stringent filters when applicable.

### Availability of supporting data

The MuTHER 450 K methylation data has been deposited in the ArrayExpress database (www.ebi.ac.uk/arrayexpress) previously, accession number E-MTAB-1866.

Processed WGBS data (minimum coverage two reads per strand and ≤ 20 % methylation difference between strands, SNPs/blacklists removed, for autosomes and chrX) for all adipose and blood samples can be visualized in the UCSC Genome Browser, (http://genome.ucsc.edu) using the Track Hub Data feature (“McGill Population Methylome”) by adding the following URL to “My Hubs”: http://hubs.hpc.mcgill.ca/~elin/McGill_TwinsUK.population.WGBS.hub.txt. All unfiltered processed WGBS data are available in the ArrayExpress database, accession number E-MTAB-3549. Raw reads from the WGBS are deposited in the European Genome-phenome Archive (EGA) and available after approval by the Data Access Committee (EGAC00001000402) designated to the study (https://www.ebi.ac.uk/ega/home) using the accession number EGAS00001001569.

## Additional files

Additional file 1: Table S1. Sequencing statistics summary. **Table S2.** Genome feature association of filtered variant-removed CpGs for adipose and blood. **Table S3.** Characteristics of unmethylated (UMR) and low-methylated regions (LMR) in adipose and blood. **Table S4.** Number of invariable sites in filtered datasets at various minimum coverages. **Table S5.** pDMR statistics in adipose and blood. **Table S6.** Result of the TFBS motif analysis using the HOMER software. **Table S7.** MZ2 vs. MZ3-derived significant DMCs overlapping known smoking methylation loci. **Table S8.** Genetic and non-genetic effect of CpG methylation. **Table S9.** Overall detected CpH methylation per tissue and strand. (XLSX 56 kb) Additional file 2: Figure S1. Number of detected CpG-sites per mean genome coverage. **Figure S2.** Overall CpG-site methylation levels. **Figure S3.** DNA methylation footprint in adipose tissue and blood. **Figure S4.** Invariable CpG tissue distribution and genomic feature association. **Figure S5.** Invariable CpG tissue distribution and genomic feature association for sites detected in ≥5 individuals. **Figure S6.** Invariable CpG tissue distribution and genomic feature association for sites detected in ≥10 individuals. **Figure S7.** Differential methylation level distribution. **Figure S8.** Genomic feature association of pDMCs. **Figure S9.** Genomic feature association of pDMRs. **Figure S10.** Genome feature association of DMCs of genetic vs. environmental origin. **Figure S11.** Proportion of eDMCs on total DMCs and eDMC genomic feature association. **Figure S12.** CpH methylation within sequence context. **Figure S13.** Overlap of methylation data with Encode blacklisted regions. **Figure S14.** CpG-site annotation scheme. **Figure S15.** pDMR definition. **Additional Figure legends**. (ZIP 1855 kb)
