# Supplementary material for: Population whole-genome bisulfite sequencing across two tissues highlights the environment as the principal source of human methylome variation
Source: Genome Biol. 2015 Dec 23;16:290. doi: 10.1186/s13059-015-0856-1 (PMC4699357; doi:10.1186/s13059-015-0856-1)

Additional Figure 1

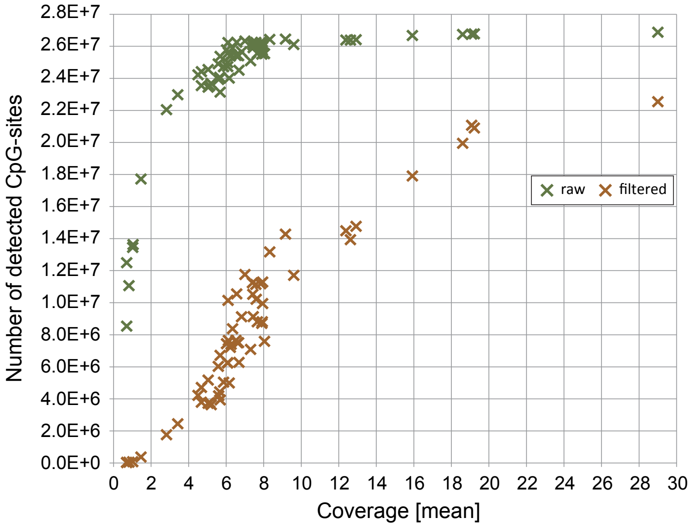

Additional Figure 2

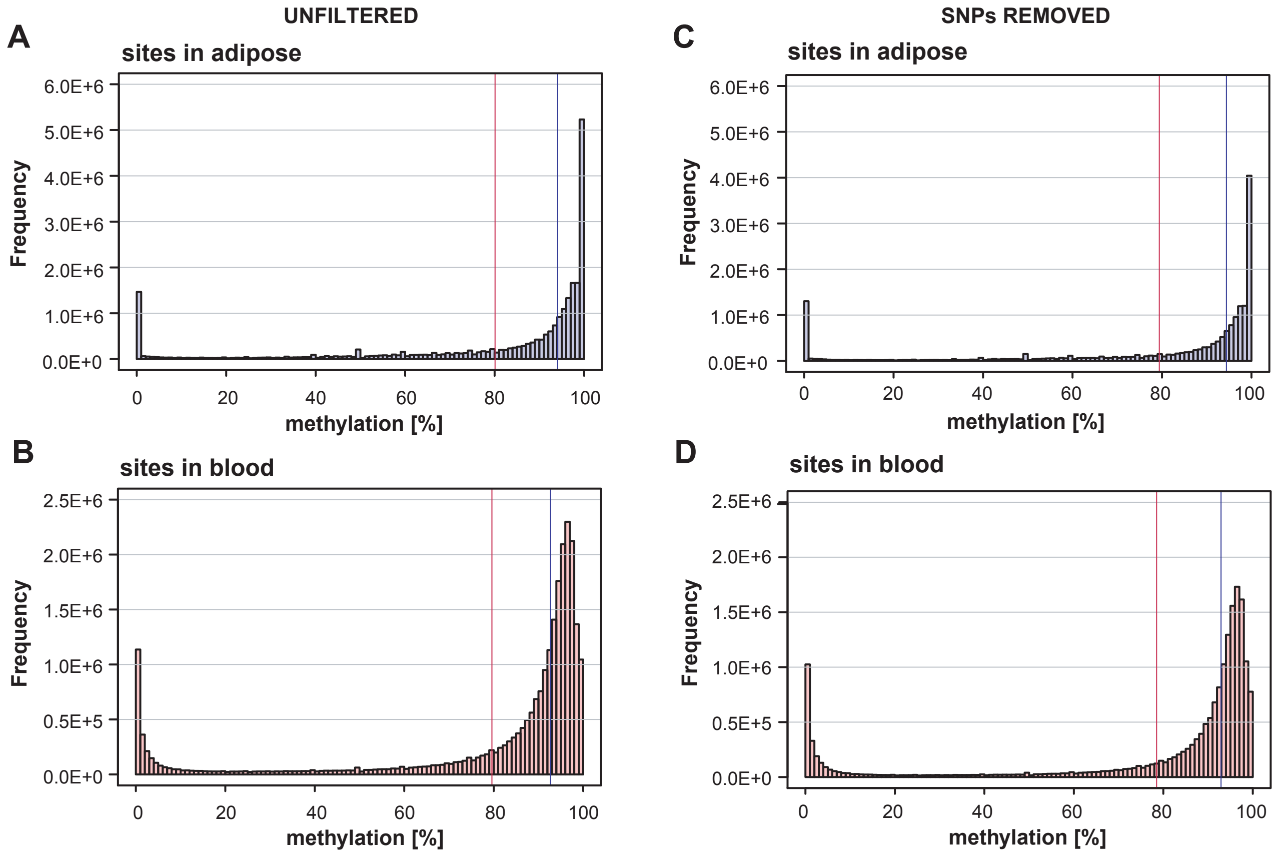

Additional Figure 3

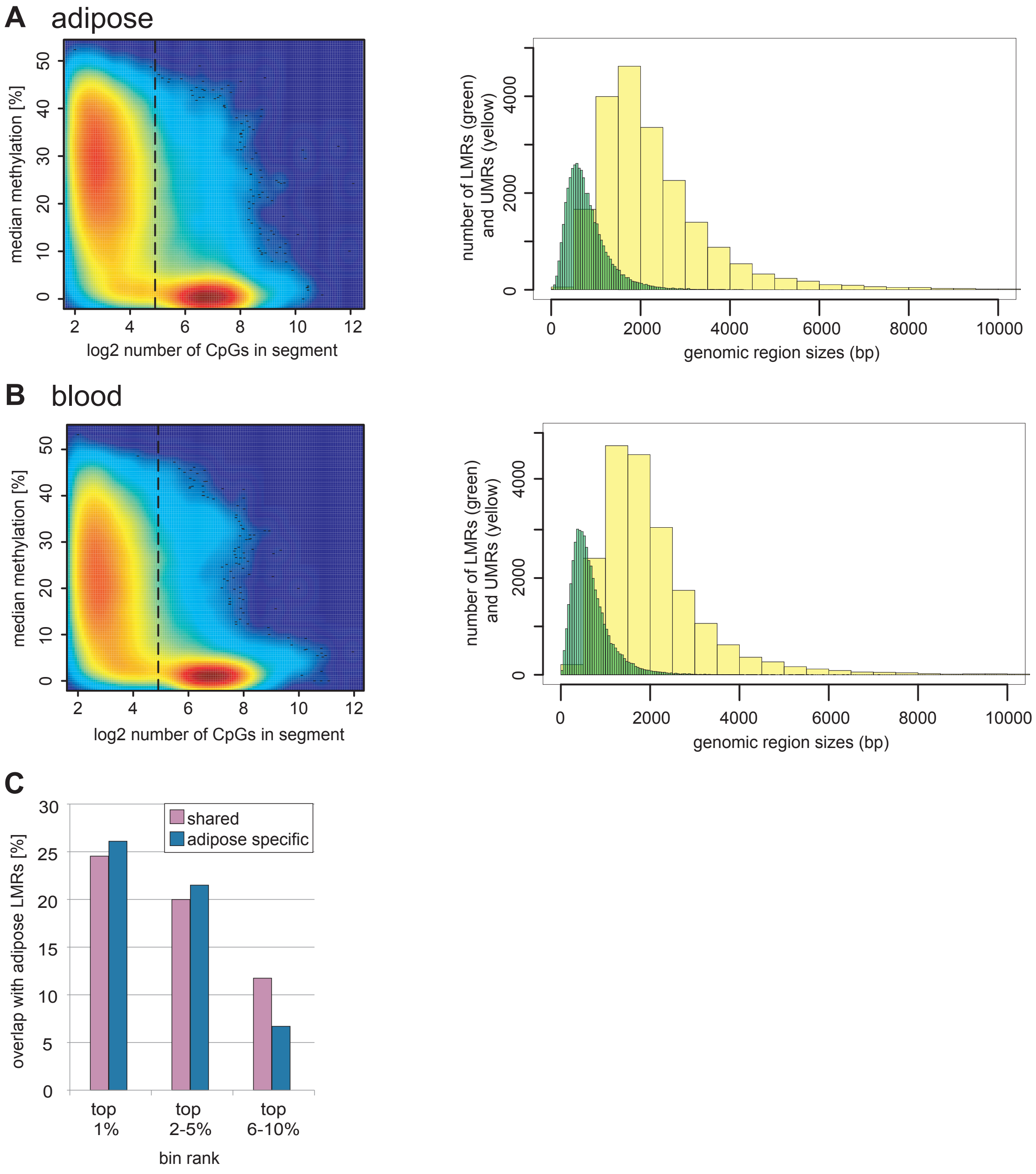

Additional Figure 4

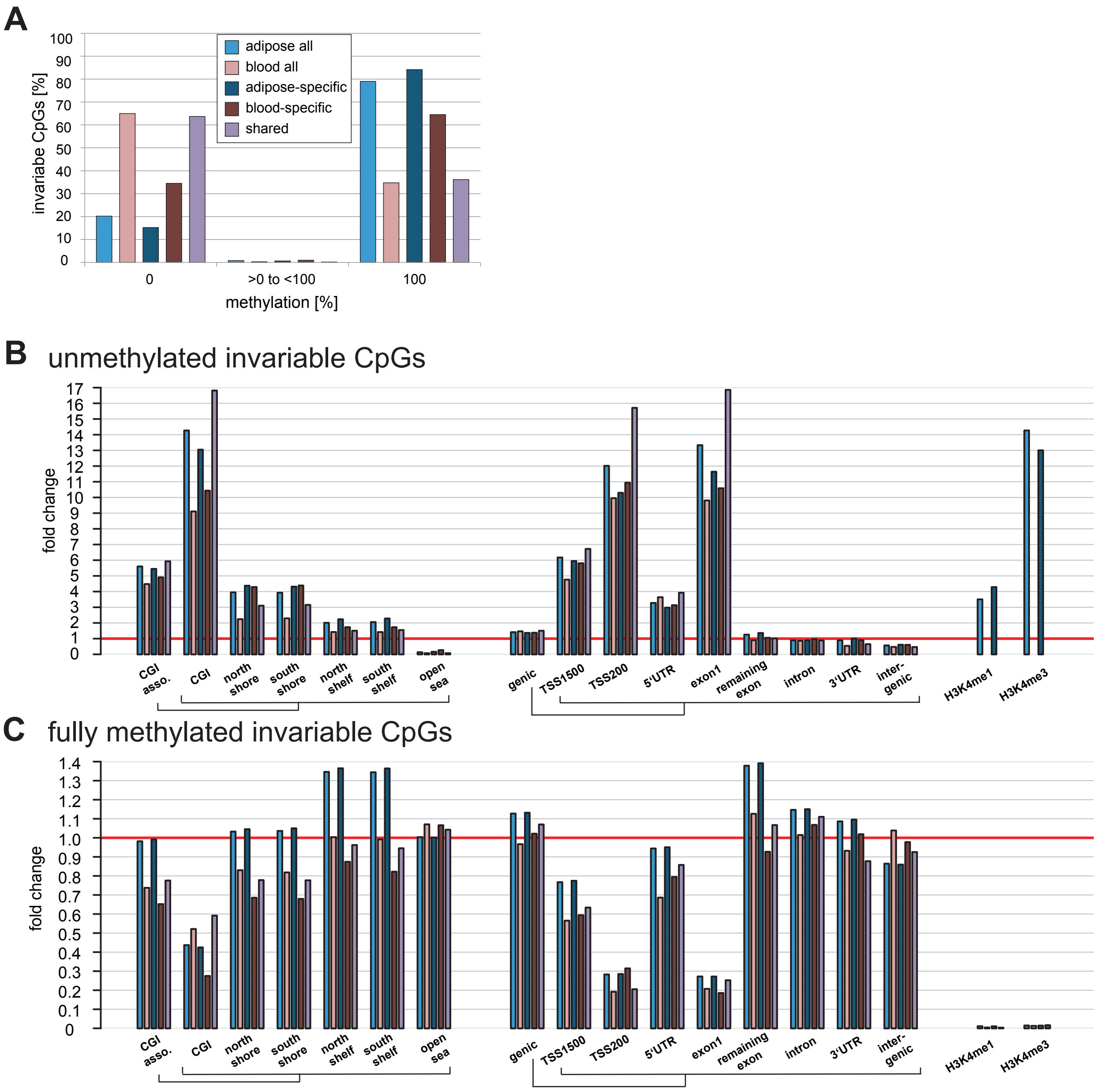

Additional Figure 5

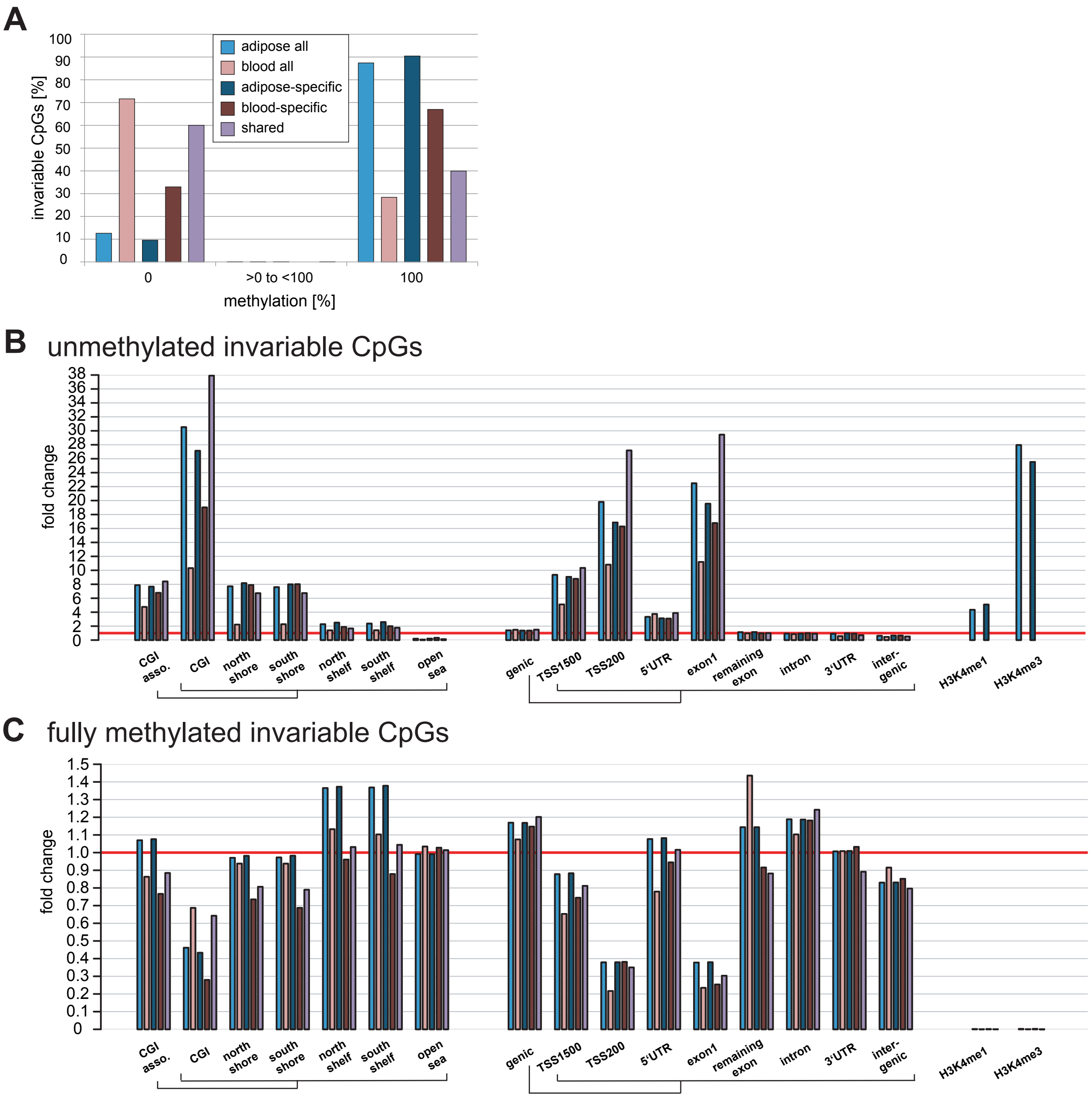

Additional Figure 6

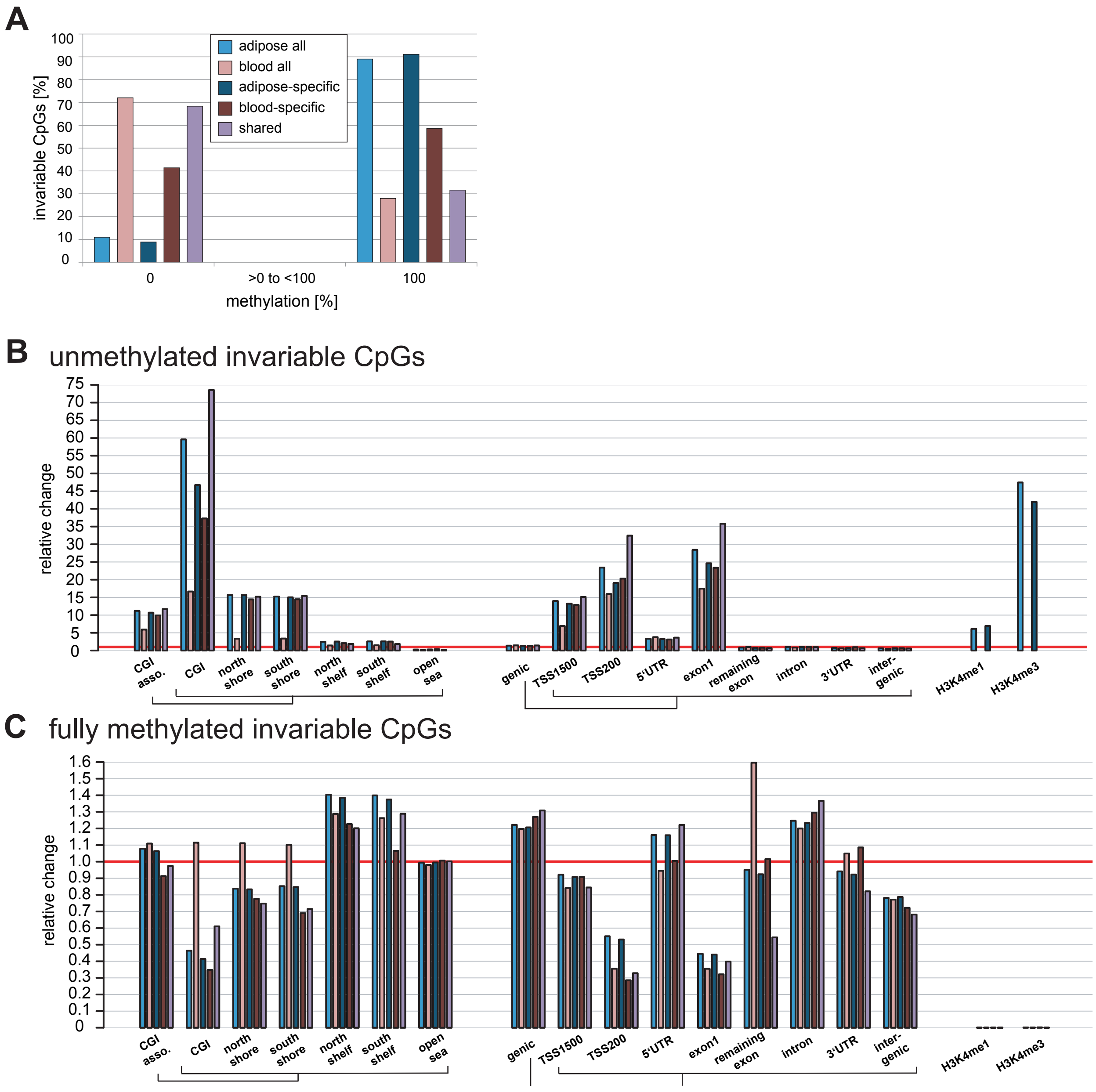

Additional Figure 7

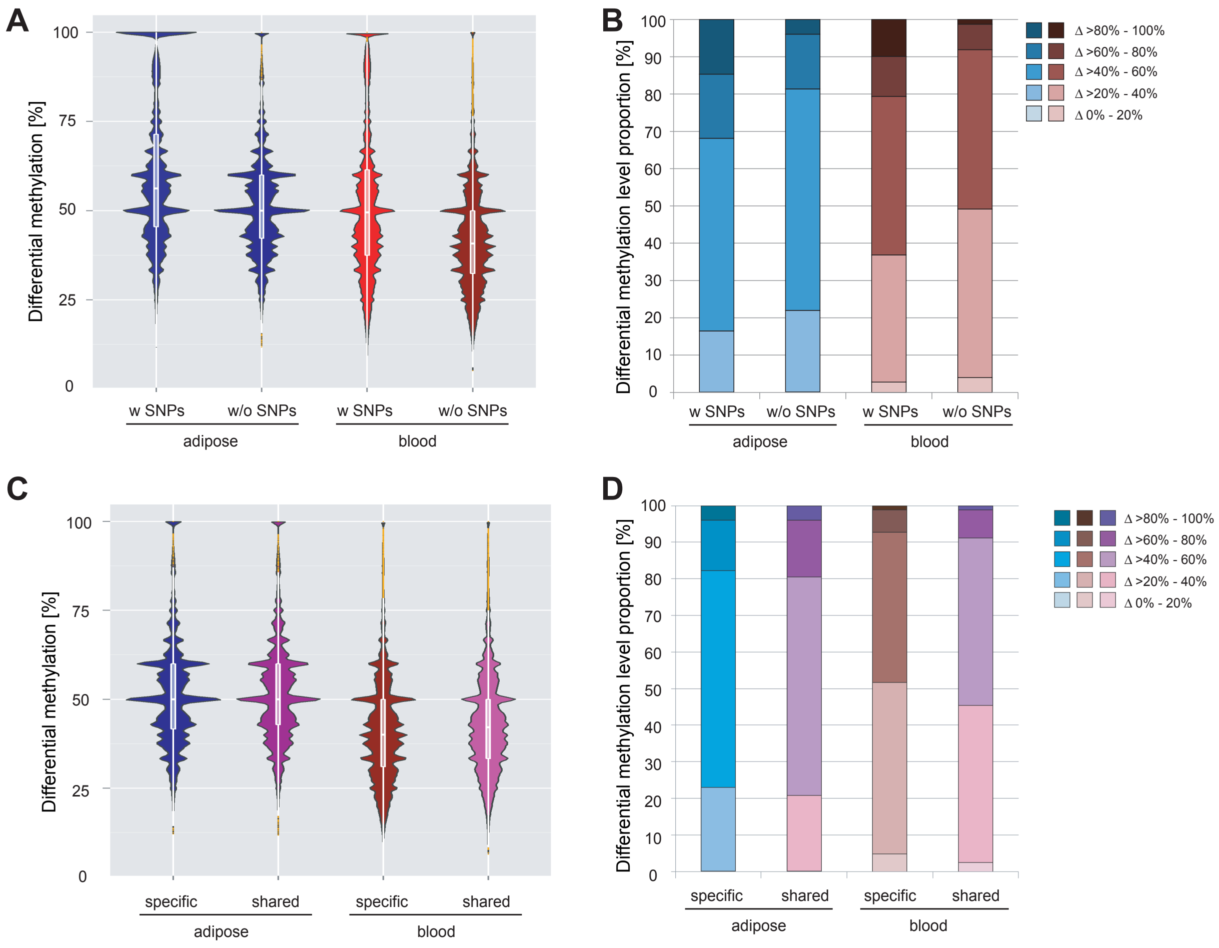

Additional Figure 8

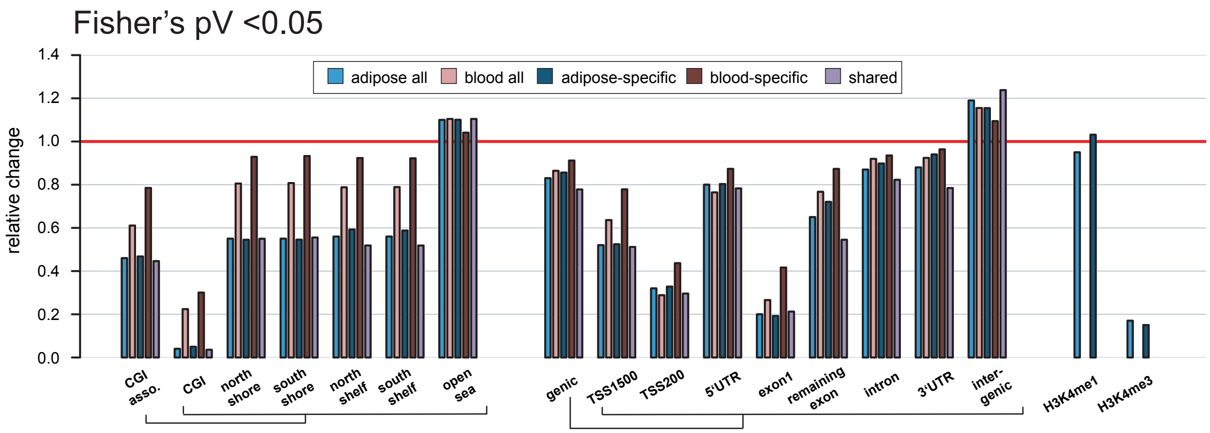

Additional Figure 9

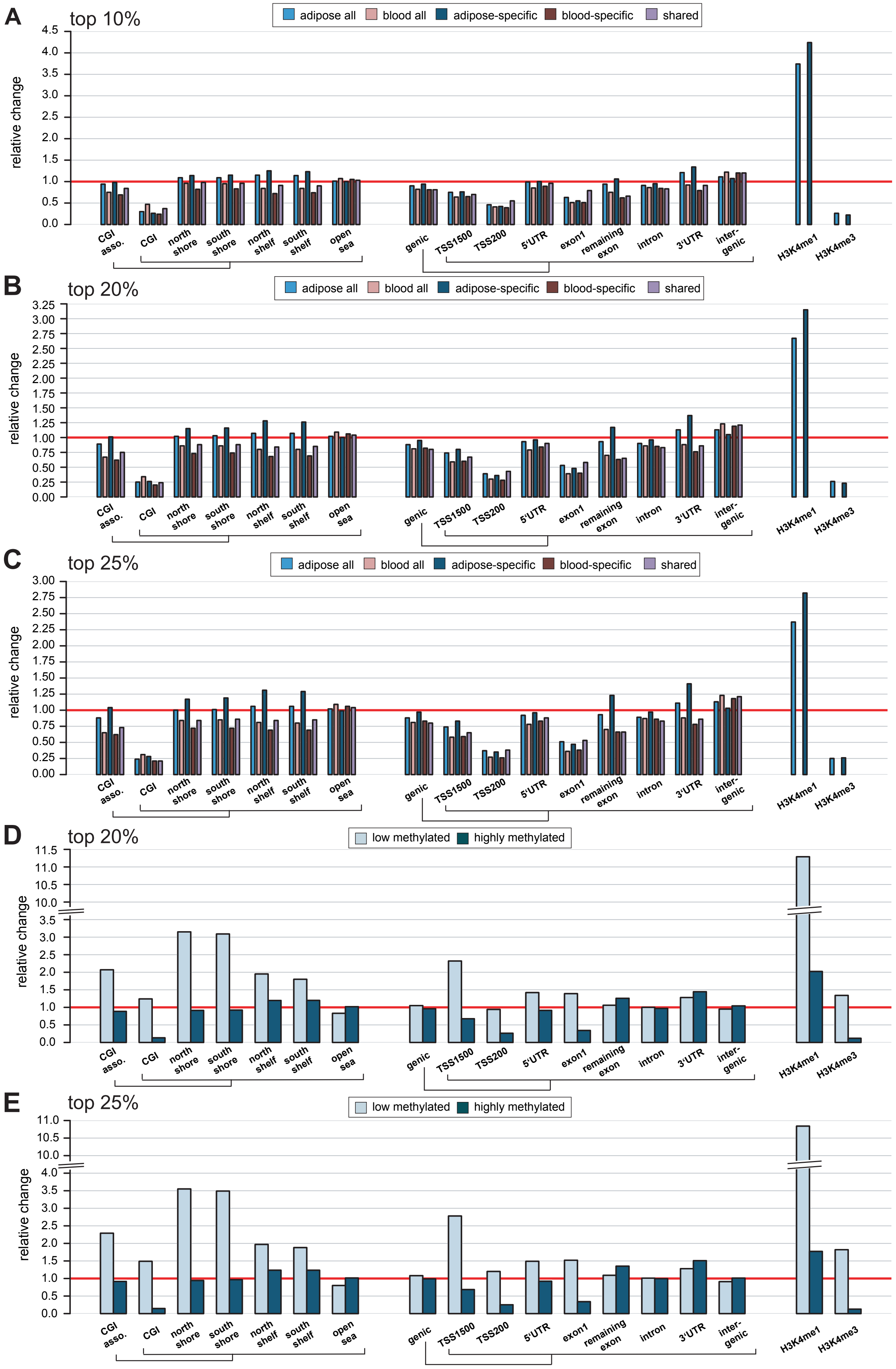

Additional Figure 10

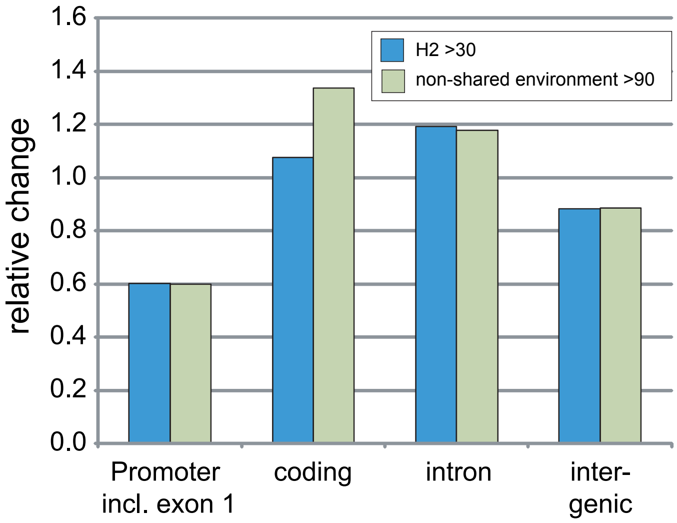

Additional Figure 11

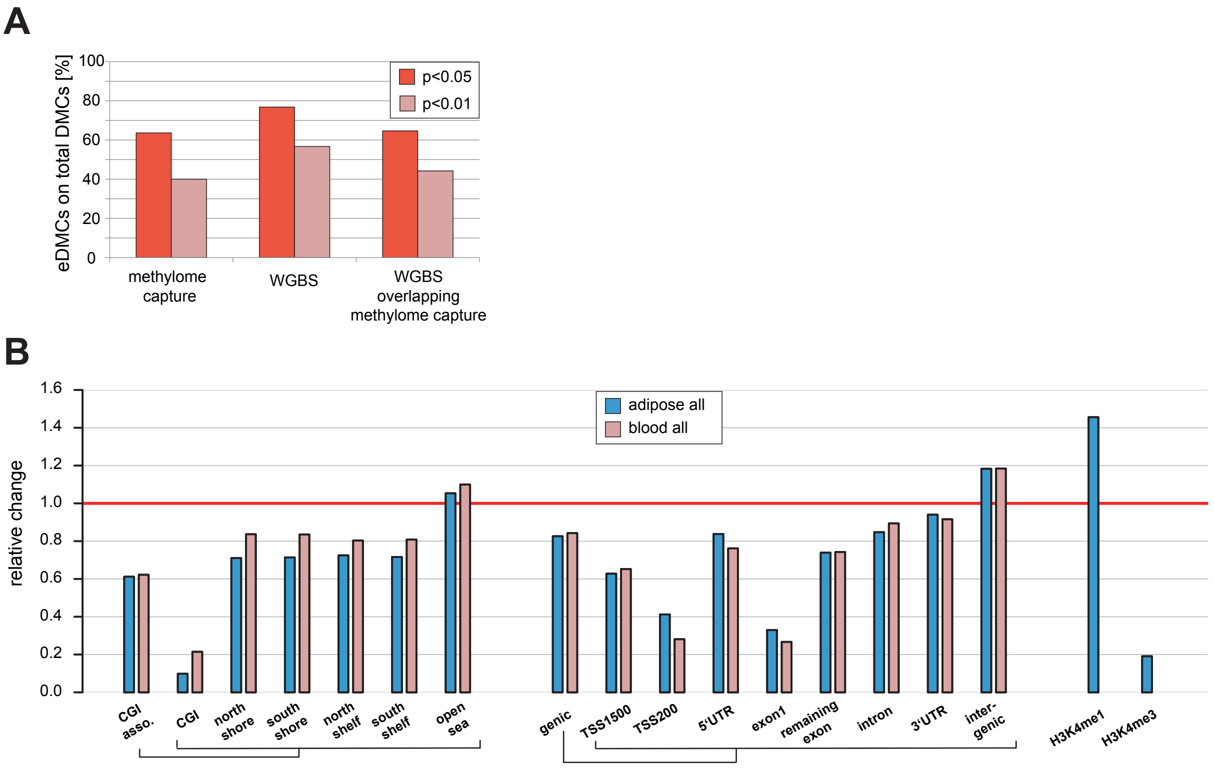

Additional Figure 12

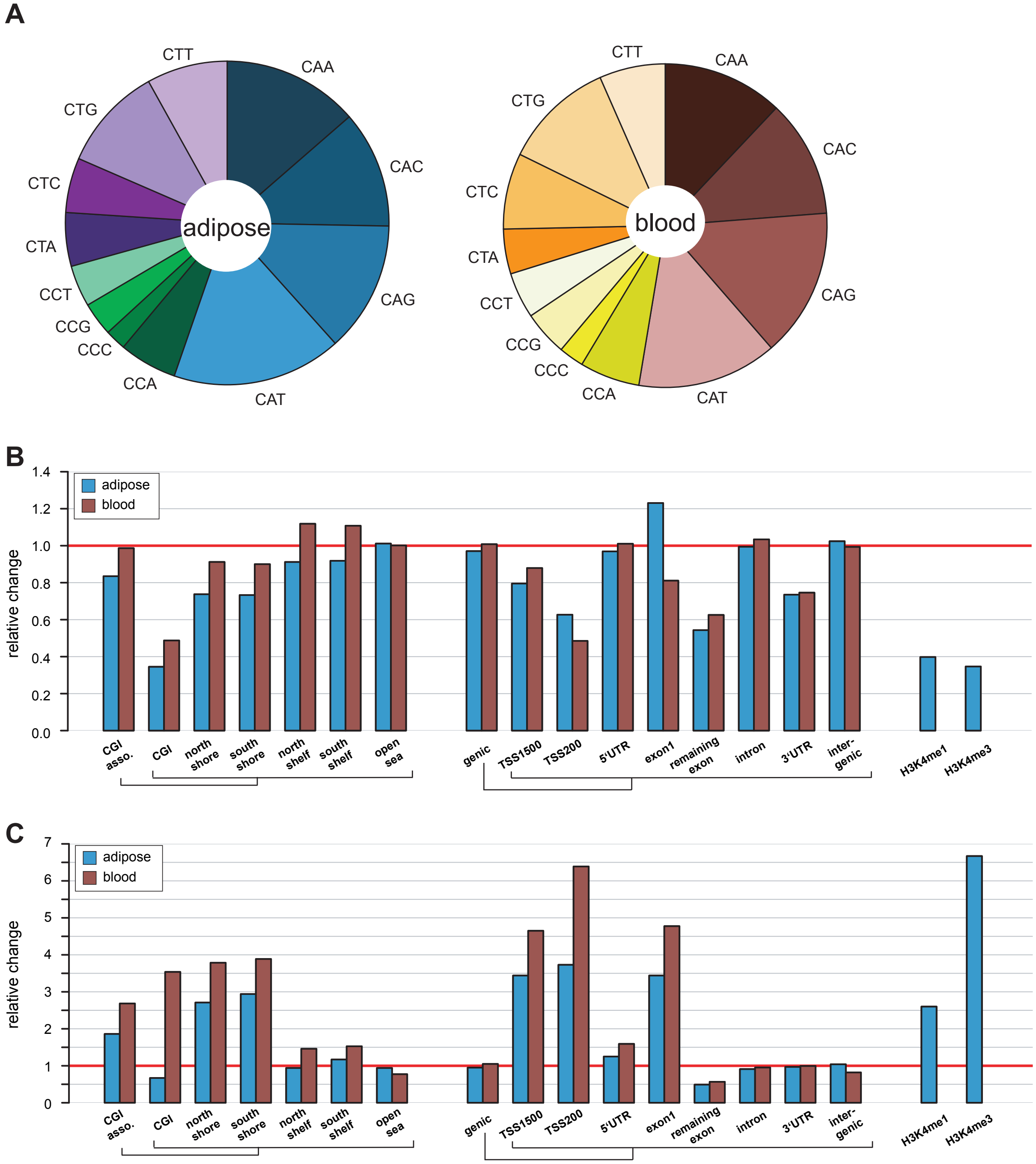

Additional Figure 13

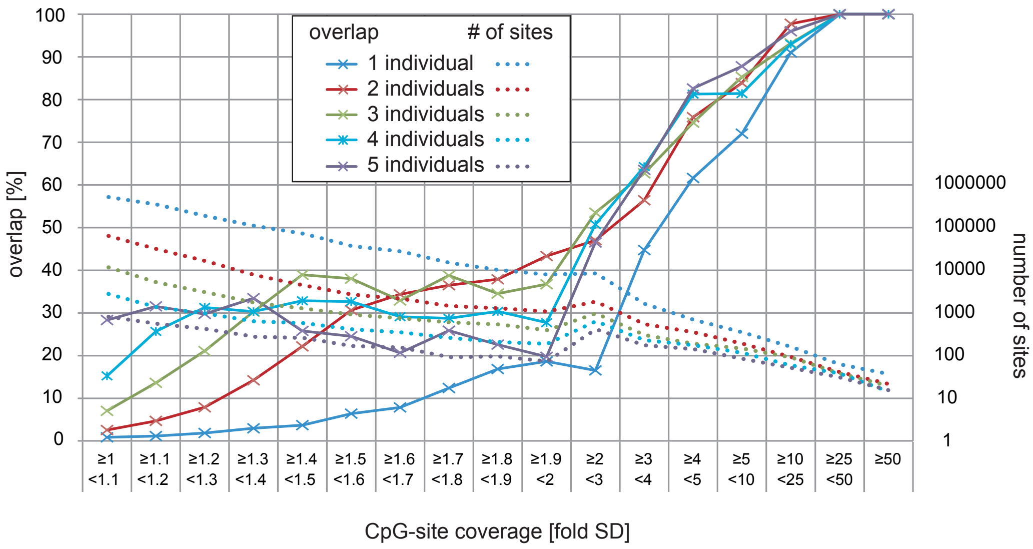

Additional Figure 14

A CpG island context

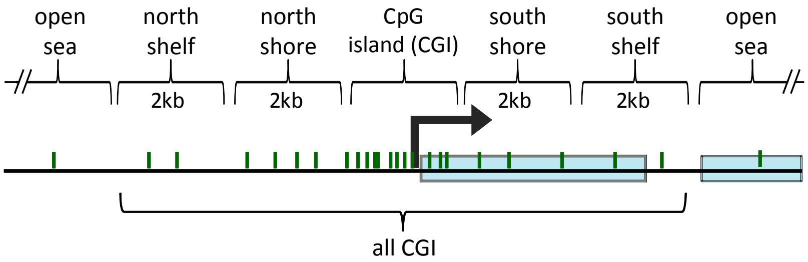

B Gene context

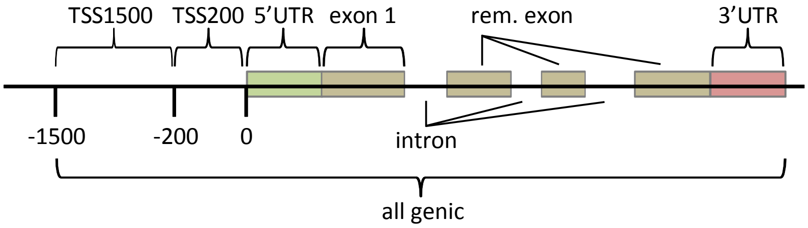

Additional Figure 15:

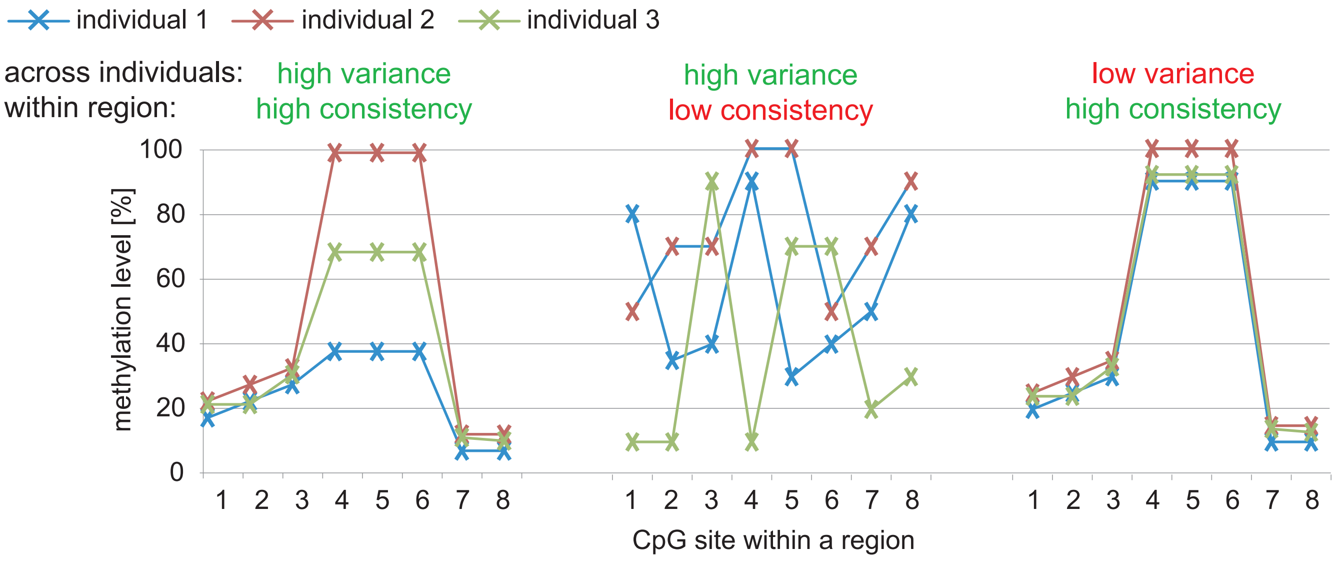

Supplement: Additional file 2: Figure S1. — Number of detected CpG-sites per mean genome coverage. Figure S2. Overall CpG-site methylation levels. Figure S3. DNA methylation footprint in adipose tissue and blood. Figure S4. Invariable CpG tissue distribution and genomic feature association. Figure S5. Invariable CpG tissue distribution and genomic feature association for sites detected in ≥5 individuals. Figure S6. Invariable CpG tissue distribution and genomic feature association for sites detected in ≥10 individuals. Figure S7. Differential methylation level distribution. Figure S8. Genomic feature association of pDMCs. Figure S9. Genomic feature association of pDMRs. Figure S10. Genome feature association of DMCs of genetic vs. environmental origin. Figure S11. Proportion of eDMCs on total DMCs and eDMC genomic feature association. Figure S12. CpH methylation within sequence context. Figure S13. Overlap of methylation data with Encode blacklisted regions. Figure S14. CpG-site annotation scheme. Figure S15. pDMR definition. Additional Figure legends. (ZIP 1855 kb) [file 13059_2015_856_MOESM2_ESM.zip › Figures_add_20151005.pdf]
