# Supplementary material for: Population whole-genome bisulfite sequencing across two tissues highlights the environment as the principal source of human methylome variation
Source: Genome Biol. 2015 Dec 23;16:290. doi: 10.1186/s13059-015-0856-1 (PMC4699357; doi:10.1186/s13059-015-0856-1)
Supplement: Additional file 2: Figure S1. — Number of detected CpG-sites per mean genome coverage. Figure S2. Overall CpG-site methylation levels. Figure S3. DNA methylation footprint in adipose tissue and blood. Figure S4. Invariable CpG tissue distribution and genomic feature association. Figure S5. Invariable CpG tissue distribution and genomic feature association for sites detected in ≥5 individuals. Figure S6. Invariable CpG tissue distribution and genomic feature association for sites detected in ≥10 individuals. Figure S7. Differential methylation level distribution. Figure S8. Genomic feature association of pDMCs. Figure S9. Genomic feature association of pDMRs. Figure S10. Genome feature association of DMCs of genetic vs. environmental origin. Figure S11. Proportion of eDMCs on total DMCs and eDMC genomic feature association. Figure S12. CpH methylation within sequence context. Figure S13. Overlap of methylation data with Encode blacklisted regions. Figure S14. CpG-site annotation scheme. Figure S15. pDMR definition. Additional Figure legends. (ZIP 1855 kb) [file 13059_2015_856_MOESM2_ESM.zip › Figures_additional_legends_20151005.docx]

**ADDITIONAL FIGURE LEGENDS**

**Additional Figure 1: Number of detected CpG-sites per mean genome coverage.**

Shown is the number of detected CpG-sites (on autosomes) detected for unprocessed data (raw; green) and for filtered sites as described in the text (filtered; brown).

**Additional Figure 2:** **Overall CpG-site methylation levels.**

Methylation levels for each CpG-site were merged across adipose and blood datasets. Histograms display the frequency of detected merged methylation levels for sites detected in (A, C) adipose and (B, D) blood for (left) filtered and (right) filtered and SNP-removed (right) datasets. Red lines indicate mean, blue lines median methylation level across the dataset.

**Additional Figure 3: DNA methylation footprint in adipose and blood.**

Adipose and blood datasets were merged within tissue keeping only sites covered ≥12-fold, and the MethylseekR software [1] was employed to identify un-and low methylated footprints. Shown is (right) the number of CpGs per unmethylated region (UMR) and low methylated region (LMR) versus its median methylation level in (A) adipose and (B) blood, with (left) histograms displaying sizes of identified LMRs (green) and UMRs (yellow) for each tissue. (C) Shared and adipose-specific LMRs were overlapped with H3K4me1 bins. Shown is the percentage of LMRs overlapping the indicated bin ranks.

**Additional Figure 4: Invariable CpG tissue distribution and genomic feature association.**

We determined invariable CpGs for each tissue (detected in ≥ 2 individuals) and distinguished between tissue-specific (methylation SD=0 in one and SD>0 in the other tissue) and tissue-shared invariable CpGs (methylation SD=0 in both tissues). (A) Shown is the methylation level distribution of invariable CpGs for each invariable CpG category (within-tissue, tissue-specific, shared). (B) Unmethylated invariable CpGs were associated with genomic features, i.e. CGI-associated and genic region and NIH Roadmap-derived H3K4me3 and H3K4me1 histone marks for adipose (for more details see text). For each genomic feature and invariable CpG category the fold change of invariable CpGs vs. CpGs in background (all CpGs detected in≥ 2 individuals) localizing to a genomic feature is shown. The red line demarks the border between enrichment (relative change > 1) and depletion (relative change < 1). (C) Same as (B) for fully methylated invariable CpGs.

**Additional Figure 5: Invariable CpG tissue distribution and genomic feature association for sites detected in ≥ 5 individuals.**

Same as Additional Figure 4 but only including sites detected in ≥ 5 individuals.

**Additional Figure 6: Invariable CpG tissue distribution and genomic feature association for sites detected in ≥ 10 individuals.**

Same as Additional Figure 4 but only including sites detected in ≥ 10 individuals.

**Additional Figure 7: Differential methylation level distribution.**

(A) Violin plots superimposed with box plots displaying differential methylation distribution of significant DMCs (Fisher’s exact test p<0.05) in adipose (blue) and blood (red) before (w SNPs) and after (w/o SNPs) confounding sequence variant removal. Upper and lower hinges in the boxplot correspond to the first and third quartiles (the 25th and 75th percentiles), upper and lower whiskers extend to the highest and lowest value that is within 1.5x the inter-quartile range of the hinge, respectively. Outliers are plotted as orange dots. (B) Bar plots showing the proportion of sites displaying between 0-20%, >20%-40%, >40%-60%, >60%-80%, >80%-100% differential methylation on the total amount of significantly differentially methylated sites for adipose and blood before and after SNP removal. (C) and (D) as (A) and (C), respectively, but for adipose-specific, blood-specific, and shared sites.

**Additional Figure 8: Genomic feature association of pDMCs.**

We determined pDMCs by carrying out pairwise comparisons of overlapping covered CpGs between samples within tissue, and then applied Fisher’s exact test to detected methylation levels at these CpGs. Significant pDMCs (p<0.05) were associated with genomic features. For each pDMC category the fold change of pDMC vs. CpGs in background (all CpGs detected in≥ 2 individuals) localizing to a genomic feature is shown. The red line demarks the border between enrichment (relative change > 1) and depletion (relative change < 1).

**Additional Figure 9: Genomic feature association of pDMRs.**

We determined pDMRs using a novel algorithm putting weight in variance of CpGs methylation across individuals as well as consistency of CpGs within the region (see methods). For each pDMR category the top (A) 10%, (B) 20%, and (C) 25% of identified pDMRs, and for adipose pDMRs subdivided into low methylated and highly methylated the top (D) 20% and (E) 25% of pDMRs were associated with genomic features as described in Additional Figure 8.

**Additional Figure 10: Genome feature association of DMCs of genetic vs. environmental origin.**

We calculated intra-class correlations (ICC) of identified adipose pDMCs that were covered in at least 5 MZ and 5 DZ adipose samples (N=4798) for the estimation of additive genetic effects, shared and non-shared environmental effects (see methods). Shown is the genome feature association for adipose pDMCs (including both adipose-specific shared pDMCs) and displaying heritability >30% (blue) and a non-shared environment of >90% (green).

**Additional Figure 11: Proportion of eDMCs on total DMCs and eDMC genomic feature association.**

(A) Employing our WGBS data we determined eDMCs by pairwise comparisons in two MZ pairs per tissue, and carried out a similar powered comparison in unrelated individuals in blood to determine the totality of blood DMCs. The same approach was carried out for methylome capture-derived DMCs generated in the same individuals, and for WGBS data overlapping locations covered in the methylome capture. Shown is the proportion of eDMCs on total DMCs detected in the methylome capture, WGBS, and for WGBS data overlapping captured regions at indicated Fisher’s exact test p-values. (B) Significant eDMCs (p<0.05) were associated with genomic features as described in Additional Figure 8.

**Additional Figure 12: CpH methylation within sequence context.**

CpH sites displaying >50% methylation and being detected in ≥2 individuals were considered methylated. (A) The pie charts illustrate the proportion of cytosine methylation within the CHH sequence context for each cytosine on total CH methylation for adipose (left) and blood (right). (B) Shown is the genomic feature association for sites displaying CpH methylation vs. CpHs in background (all CpHs detected in≥ 2 individuals) for adipose and blood. The red line demarks the border between enrichment (relative change > 1) and depletion (relative change < 1). (C) Genomic feature association of highCpH-lowCpG methylation at individual level against background (all detected CpH sites) as in (B).

**Additional Figure 13: Overlap of methylation data with Encode blacklisted regions.**

For each sample mean genome coverage and standard deviation (SD) was determined. Shown is the percentage of sites overlapping with DBRs and DERs being covered at the indicated amount of individuals at a given coverage interval (x-fold the SD). Dotted lines indicate the number of CpG-sites within indicated coverage intervals.

**Additional Figure 14: CpG-site annotation scheme.**

Each CpG-site was annotated within (A) the CpG-island (CGI) context to be located within a CGI, within a CGI shore (2kb upstream (north) or downstream (south) of a CGI), within a CGI shelf (2kb upstream (north) or downstream (south) of a CGI shore), or, if not in any of above to be located in open sea; (B) the gene context to be located within the TSS1500 (1.5kb to 200bp upstream of a transcription start site (TSS)), TSS200 (200bp upstream of a TSS), the 5’UTR, exon 1, the remaining exons (rem. exons), introns, the 3’UTR, or, if in none of the above, to be intergenic.

**Additional Figure 15: pDMR definition.**

We determined pDMRs by putting weight in variance of CpG methylation across individuals as well as consistency of CpGs within the region. The diagram shows a theoretical example of methylation levels detected at eight CpG-sites in 3 individuals. Only the example on the left fulfills our pDMR selection criteria displaying high consistency within region and high variance across individuals. Examples in the middle and right are not classified as pDMRs as they display low consistency within region or low variance across individuals, respectively.

**REFERENCES:**

1. Burger L, Gaidatzis D, Schubeler D, Stadler MB: **Identification of active regulatory regions from DNA methylation data.** *Nucleic Acids Res* 2013, **41:**e155.

2. Heinz S, Benner C, Spann N, Bertolino E, Lin YC, Laslo P, Cheng JX, Murre C, Singh H, Glass CK: **Simple combinations of lineage-determining transcription factors prime cis-regulatory elements required for macrophage and B cell identities.** *Mol Cell* 2010, **38:**576-589.
